# Novel Insights into circRNA Saga Coming from Spermatozoa and Epididymis of HFD Mice

**DOI:** 10.3390/ijms24076865

**Published:** 2023-04-06

**Authors:** Francesco Manfrevola, Teresa Chioccarelli, Vincenza Grazia Mele, Veronica Porreca, Monica Mattia, Donatella Cimini, Antonella D’Agostino, Gilda Cobellis, Silvia Fasano, Chiara Schiraldi, Rosanna Chianese, Riccardo Pierantoni

**Affiliations:** 1 Department of Experimental Medicine, University of Campania “Luigi Vanvitelli”, 80138 Naples, Italy; 2 Department of Environmental, Biological and Pharmaceutical Sciences and Technologies, University of Campania “Luigi Vanvitelli”, 81100 Caserta, Italy

**Keywords:** circRNAs, high-fat diet, spermatozoa, male infertility, oxidative stress

## Abstract

Obesity is a pathophysiological disorder associated with adiposity accumulation, oxidative stress, and chronic inflammation state that is progressively increasing in younger population worldwide, negatively affecting male reproductive skills. An emerging topic in the field of male reproduction is circRNAs, covalently closed RNA molecules produced by backsplicing, actively involved in a successful spermatogenesis and in establishing high-quality sperm parameters. However, a direct correlation between obesity and impaired circRNA cargo in spermatozoa (SPZ) remains unclear. In the current work, using C57BL6/J male mice fed with a high-fat diet (HFD, 60% fat) as experimental model of oxidative stress, we investigated the impact of HFD on sperm morphology and motility as well as on spermatic circRNAs. We performed a complete dataset of spermatic circRNA content by a microarray strategy, and differentially expressed (DE)-circRNAs were identified. Using a circRNA/miRNA/target network (ceRNET) analysis, we identified circRNAs potentially involved in oxidative stress and sperm motility pathways. Interestingly, we demonstrated an enhanced skill of HFD sperm in backsplicing activity together with an inefficient epididymal circRNA biogenesis. Fused protein in sarcoma (FUS) and its ability to recruit quaking (QKI) could be involved in orchestrating such mechanism.

## 1. Introduction

In recent years, the field of metabolic diseases linked to overweight conditions has acquired considerable importance as obesity is linked to a set of pathophysiological disorders, such as systemic oxidative stress/inflammation, insulin resistance, liver steatosis, and abdominal adiposity accumulation [1,2,3,4,5,6,7].

Considering that, in the last decades, the onset of metabolic syndrome has undergone a strong acceleration coincident with the reproductive age, a compromised ability to successfully achieve pregnancy, due to affected male reproductive skills dependent on obesity and oxidative stress conditions, has been well established [8,9]. In fact, the study of male mice models, chronically fed with high-fat diets (HFD mice) to mimic a metabolic syndrome, allowed for identifying several impaired testicular and spermatic functions, such as: (i) increased testicular cholesterol content; (ii) alteration of spermiogenesis; (iii) interrupted integrity of the blood–testis barrier; (iv) testicular germ cell apoptosis; (v) decline of testosterone production; (vi) defective acrosome biogenesis; (vii) low sperm production; (viii) altered morphology of the sperm head; and (ix) decrease of sperm motility [10,11,12]. In this regard, oxidative stress increases poor sperm quality parameters as: (i) DNA double-stranded break damage; (ii) chromatin remodeling defects; and (iii) mitochondrial dysfunctions that negatively impact sperm motility [13,14,15,16].

Currently, there is a growing concern regarding the deregulation of noncoding RNAs (ncRNAs) in metabolic diseases, oxidative stress, and male infertility onset; indeed, an intrinsic involvement of a dysregulated expression of ncRNAs in metabolic disorders, including insulin resistance and type 2 diabetes, oligozoospermia, and sperm anomalies, has been clarified [17,18,19,20,21,22]. Accordingly, lncRNA sequencing data derived from oligozoospermic sperm have highlighted a robust set of differentially expressed (DE)-lncRNAs mainly implicated in unfolded proteins, oxidative stress, and sperm cell apoptotic pathways, potentially involved in oligozoospermia and sperm anomalies [22].

CircRNAs are a class of ncRNAs that participate in the post-transcriptional regulation of gene expression by a tethering activity exerted on miRNAs in order to protect the related mRNA targets from degradation. The weakening of miRNA effects on mRNA translation, dependent on circRNA sponge activity, makes possible that circRNAs take part in a competitive endogenous RNA (ceRNA) network (ceRNET) able to regulate gene expression [23,24]. CircRNAs are covalently closed RNA molecules produced by backsplicing, a molecular mechanism during which a downstream (3′) splice donor site is covalently linked to an upstream (5′) splice acceptor site to produce the circular molecules [24,25]. Canonical spliceosomal machinery may be employed in linear splicing as well as in backsplicing, but the success of circRNA formation is guaranteed by specific RNA-binding proteins (RBPs), including RNA polymerase II (RNApol2), quaking (QKI), and fused protein in sarcoma (FUS), which actively orchestrate this intricate molecular mechanism [26,27,28,29]. Several types of circRNAs have been discovered and classified, but the most abundant in cells consist in exonic circRNAs, a class of circRNAs promoted by inverted repeats in cis-flanking sequences and preferentially located in the cytoplasm. Exonic circRNA biogenesis is also tightly dependent on trans-factors as RBPs; indeed, binding sites for FUS and QKI occur in the flanking intron regions [26,27,28,29]. The lack of poly-A tails and 5′ caps in circRNAs, due to their covalently closed structure, allows such molecules to escape from several canonical RNA decay pathways inducing cellular circRNA accumulation due to their high stability [27]. 

CircRNAs are acquiring an increasingly prominent role in male fertility and sperm quality setting; indeed, circRNAs have been identified in human and mouse testis, expressed in spermatogenic cells, and finally correlated with germ cell progression and sexual development [30,31,32]. An endogenous skill to circularize mRNAs has been characterized in murine and human spermatozoa (SPZ) [29]. In addition, spermatic circRNAs have been identified in human SPZ and appear differentially expressed in relation to sperm morphological quality parameters, as well as to subcellular localization [33]. In this scenario, a new potential application of circRNAs as biomarkers of sperm quality has been highlighted by profiling a complete dataset of circRNA cargo in asthenozoospermic patients [34], and not least, a new intriguing role of circRNAs in the modulation of sperm nuclear remodeling dynamics, during their epididymal maturation, has been recently reported [35]. 

Despite the consolidated role of circRNAs and body weight condition in the control of the optimal spermatogenesis process, as well as in the regulation of male reproductive functions, a rigorous study concerning an alleged functional link between metabolic disorders, oxidative stress, and related molecular effects on spermatic circRNAs has never been performed. 

In the current work, in order to shed light on a putative association between obesity and spermatic circRNA cargo, we carried out a fine microarray analysis on total epididymal SPZ collected from control (CTRL) and HFD mice. Spermatic DE-circRNAs were identified, and an anomalous backsplicing mechanism in both HFD SPZ and epididymis was highlighted. Then, in order to suggest that DE-circRNAs may be key regulators of HFD altered sperm motility, we carried out a vesicle shuttle in vitro experiment focused on a circSMAD2-dependent regulatory pathway. 

## 2. Results

### 2.1. General Features of HFD Model

C57BL/6 male mice were fed with HFD (60% fat) or CTRL diet for a time of 12 weeks starting at 8 weeks of age. Body weight was assessed once weekly in the two experimental groups (Figure S1A). During the feeding period, HFD mice rapidly increased their rate of weight gain starting at 11 weeks of age and continuing until the end of treatment, which coincided with 20 weeks of age (Figure S1A). Additionally, at the end of treatment, the size of HFD was greater than that of CTRL mice (Figure S1B). In detail, a significant increase in body weight (*p* < 0.01) was observed in HFD as compared with CTRL mice (Figure S1C). No significant changes were observed in the body length of both experimental groups (Figure S1D), while a significant increase in abdominal circumference (*p* < 0.01) occurred in HFD mice (Figure S1E).

In order to investigate HFD effects on organs and tissues, we carried out systematic weight measurements and morphological analysis in the three main districts reported to be affected in HFD condition: liver, heart, and testis. As reported in Table 1, a significant increase in weight for the liver (1.57 ± 0.02 vs. 1.38 ± 0.03; *p* < 0.01) and heart (0.29 ± 0.04 vs. 0.20 ± 0.02; *p* < 0.05) was observed in HFD mice, while no significant changes occurred in testis weight. Morphological analysis carried out by hematoxylin and eosin (H&E) staining showed a clear hepatic steatosis and fat vacuole accumulation in the hepatic cells of HFD (Figure S1F). In addition, the morphological analysis of longitudinal sections of heart tissue highlighted a cardiac hypertrophy in HFD in comparison with CTRL heart (Figure S1F), as confirmed by the significant increase in cardiomyocyte diameter (*p* < 0.01) observed in HFD heart sections (Figure S1G). 

To evaluate the metabolic condition of HFD mice, the dosage of serum lipids, insulin, leptin, and blood glucose was carried out. As reported in Table 2, data showed significantly higher levels of all measured parameters in HFD as compared with CTRL mice, clearly indicating a hyperlipidemic, hyperinsulinemic, and hyperleptinemic phenotype in HFD mice. 

### 2.2. Impact of HFD on Testis and Sperm Morphological and Functional Parameters

**Figure 1:**
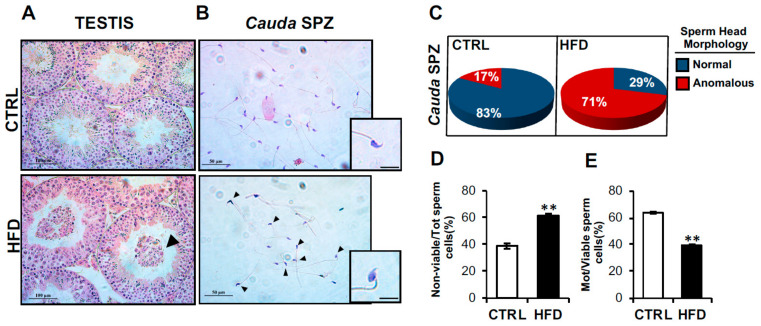
**Characterization of HFD testis and sperm parameters.** (**A**,**B**) H&E staining of testis (**A**) and *cauda* SPZ (**B**) collected from CTRL (n = 6) and HFD (n = 6) mice. (**A**) In testis sections, germ cell lumen infiltration was indicated by black arrowheads; scale bar: 100 μm. (**B**) Anomalous sperm heads were indicated by black arrowheads; scale bar: 50 μm; scale bar inset: 10 μm. (**C**) Percentage of anomalous sperm heads in CTRL and HFD *cauda* SPZ; data were reported as the percentage of anomalous sperm heads/total SPZ. Sperm viability (**D**) and motility (**E**) assay in CTRL and HFD *cauda* SPZ; data were expressed as the percentage of nonviable/total SPZ and motile/live SPZ, respectively, and reported as mean ± SEM; ** *p* < 0.01.

To assess the impact of HFD on testis and sperm fertilizing ability, we carried out a morphological analysis of testis as well as both morphological and functional analyses of *cauda* SPZ collected from CTRL and HFD mice. Testicular morphological evaluation carried out by H&E staining showed a disorganized seminiferous epithelium in HFD testis, characterized by a severe germ cell lumen infiltration, dependent on their detachment from seminiferous epithelium (Figure 1A). H&E staining of CTRL and HFD *cauda* SPZ, which represent the spermatic population with the highest maturation grade and therefore having fertilizing capacity, showed a large number of HFD SPZ with structural defects in sperm head (Figure 1B), suggesting that an anomalous development of head shaping occurred in HFD mice. Accordingly, the count of total SPZ with anomalous sperm head morphology highlighted a higher percentage of sperm head defects (71%) in HFD than CTRL (17%) mice (Figure 1C). 

Based on these observations, we investigated sperm functional parameters, as viability and motility, in order to assess if HFD affected sperm reproductive skills. As reported, the number of nonviable sperm cells was significantly higher (*p* < 0.01) in HFD than CTRL SPZ (Figure 1D), while a drastic reduction of motility (*p* < 0.01) occurred in HFD SPZ (Figure 1E), confirming that HFD negatively affected sperm viability and motility. 

### 2.3. CircRNA Expression Profile in CTRL and HFD Epididymal SPZ 

**Figure 2:**
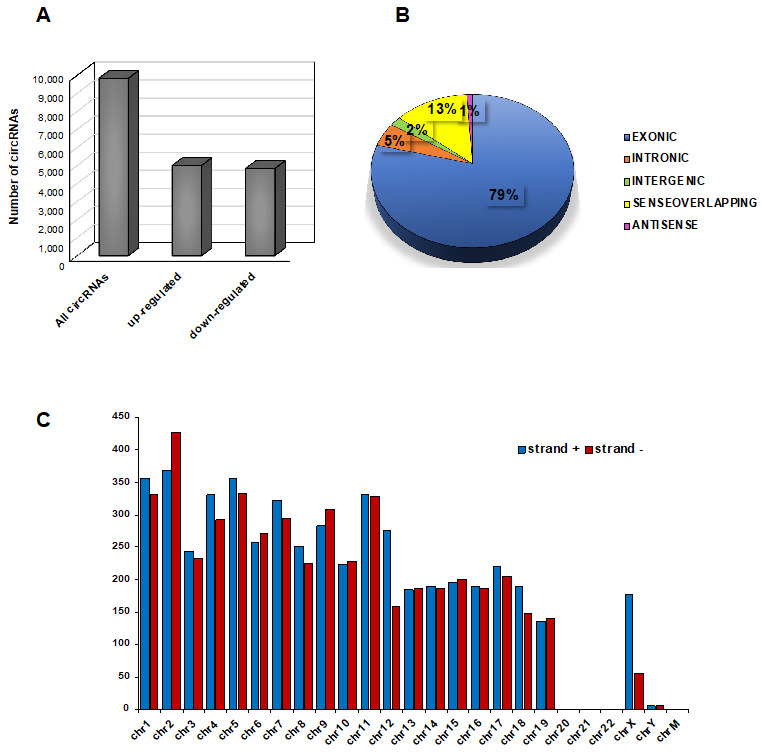
**Overview of circRNA expression in mouse SPZ.** (**A**) The distribution of up- and downregulated circRNAs in HFD (n = 3) compared with CTRL (n = 3) SPZ among a total of 9838 circRNAs. (**B**) The proportion of different types of circRNAs among all predicted circRNAs. (**C**) Chromosomal distribution of SPZ-derived circRNAs on strand + and strand –, according to their host gene location.

CircRNAs’ profile was investigated in epididymal SPZ collected from CTRL (n = 3) and HFD (n = 3) male mice by using microarray analysis. We identified in epididymal murine SPZ a total of 9838 circRNAs that appeared up- (n = 5003) or downregulated (n = 4835) in HFD SPZ (Figure 2A). The identified circRNAs were clustered according to their structure. In detail, exonic circRNAs represented the most abundant type of spermatic circRNA (78.6%), followed by sense overlapping (12.6%) and, finally, by intronic (5.1%), intergenic (2.1%), and antisense (1.6%), which appeared the less represented in epididymal SPZ (Figure 2B). According to the analysis of circRNA host genes, circRNAs were principally generated by chromosomes 1 and 2. Interestingly, only few circRNAs were derived from chromosomes 19, X, and Y, while chromosomes 20, 21, 22 and mitochondrial (M) did not produce any circRNA. In addition, the majority of circRNAs derived from strand + were dependent on host genes distributed across chromosomes 1, 4, 5, 7, 12, 17, 18, and X, while a reversed profile was observed for the other ones distributed on chromosomes 2, 6, and 9 (Figure 2C). 

### 2.4. Identification of DE-circRNAs in HFD vs. CTRL Epididymal SPZ 

**Figure 3:**
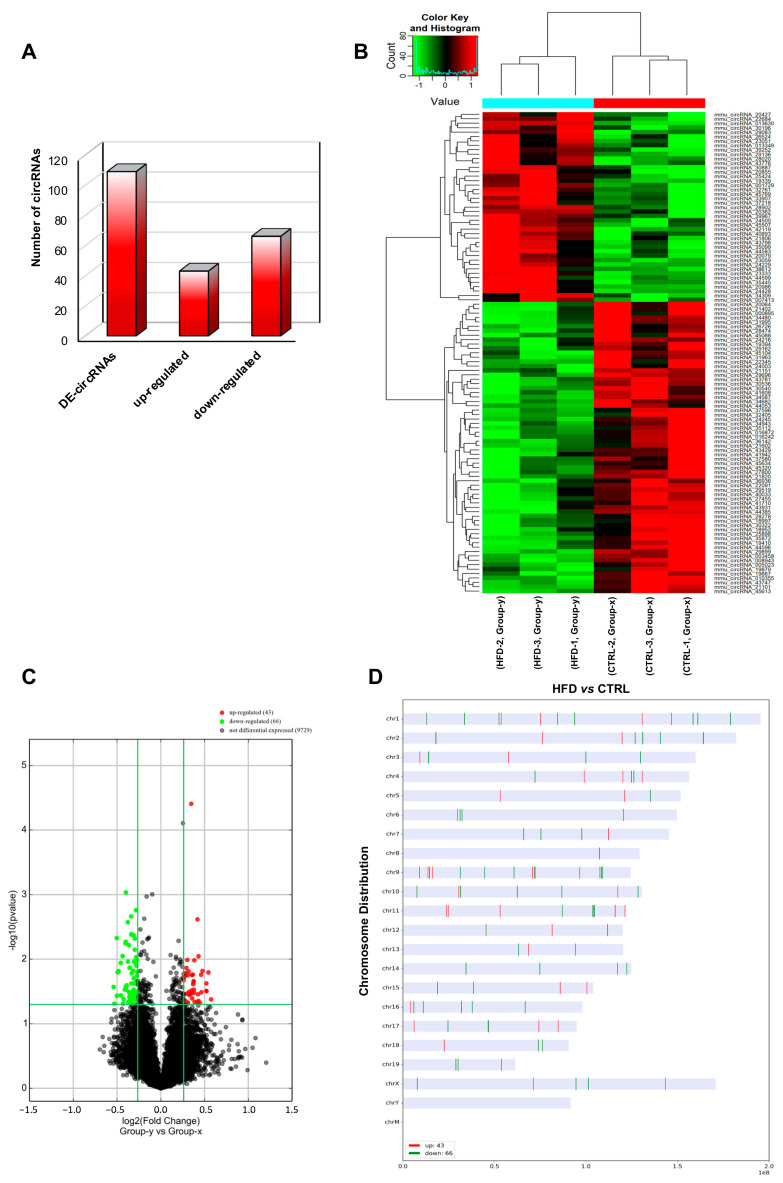
**Differential expression of circRNAs between CTRL and HFD SPZ.** (**A**) The distribution of up- and downregulated DE-circRNAs, among a total of 109 DE-circRNAs, in HFD compared with CTRL SPZ. (**B**) Hierarchical clustering analysis of DE-circRNAs in CTRL SPZ (samples CTRL-1, CTRL-2, CTRL-3) and HFD SPZ (samples HFD-1, HFD-2, HFD-3); the expression values (fold change > 1.5, *p* < 0.05) were represented in different colors, indicating expression levels above and below the median expression level across all samples. (**C**) The volcano plot was constructed using fold change and *p*-values; in detail, the values on X and Y axes are log2 (FC = fold change) and –log10 (*p*-values), respectively. Red points in the volcano plot represent the DE-circRNAs with statistical significance. (**D**) The distribution of up- and downregulated DE-circRNAs in the mouse genome, according to their host gene location.

In order to perform a finer analysis of circRNA microarray data, we chose stringent parameters, such as fold change cut-off >1.2 and *p*-values cut-off <0.05, able to identify DE-circRNAs in HFD SPZ. As reported, a total of 109 DE-circRNAs, clustered in 43 upregulated and 66 downregulated, were identified in HFD SPZ (Figure 3A). Heatmap and volcano plot analyses were used to represent DE-circRNAs clustered by fold change and the variations of circRNA expression profiles between HFD and CTRL SPZ, respectively (Figure 3B,C). The distribution of DE-circRNAs was analyzed depending on the genome location of their host gene. Despite that an equal number of both up- and downregulated DE-circRNAs were distributed across almost all chromosomes, most of the circRNAs downregulated in HFD SPZ were generated by chromosomes 2, 9, 10, and 16, while chromosome 8 contained only downregulated ones. Differently, this profile was reversed on chromosome 11, and accordingly, chromosomes 20, 21, 22, Y, and M did not show circRNAs (Figure 3D). 

### 2.5. Functional Clustering, Gene Ontology (GO), and KEGG Analysis of DE-circRNAs

The association between DE-circRNAs and related miRNA targets was predicted by using bioinformatic tools in order to build spermatic functional ceRNETs (Figure S2). The same behavior was highlighted for both circRNAs up- and downregulated in HFD when compared with CTRL SPZ. In detail, tethering activity toward the same miRNA (rectangular symbol) was exerted by different circRNAs (ovoid symbols), which could derive from host genes located on the same or different chromosomes (Figure S2A,B). 

GO functional enrichment and KEGG pathway analyses were carried out to define the biological processes and molecular pathways, respectively, in which DE-circRNAs up- and downregulated in HFD SPZ were involved (Figures S3 and S4, respectively). The GO top results, clustered in three main areas (biological process, cellular component, and molecular function), showed that DE-circRNAs were involved in biological processes, such as glucose catabolic process, cAMP metabolic process, and developmental growth. The most significant *p*-values of up- and downregulated DE-circRNAs in HFD SPZ were presented in the bar blot (Figure S3A,B, respectively). KEGG pathway results showed that circRNAs upregulated in HFD SPZ were linked to metabolic pathways, including ovarian steroidogenesis, thermogenesis, thyroid hormone synthesis, and regulation of lipolysis in adipocytes (Figure S4A), while cell cycle and TGF-beta signaling were the principal pathways linked to circRNAs upregulated in HFD SPZ (Figure S4B). 

Considering that circRNAs are able to harbor several miRNAs, we carried out the construction of a ceRNET for one representative circRNA, up- and downregulated in HFD SPZ, in order to shed light on predicted mRNA targets and their potential involvement in the oxidative stress pathway. Among circRNAs upregulated in HFD SPZ, we chose circADAM10 since its linear transcript encodes for a metalloproteinase needed to prevent neuroinflammation and oxidative stress [36], while circPCSK6 was chosen as representative circRNA downregulated in HFD SPZ as recently reported to be involved in the prevention of senescence phenotype and cellular dysfunction dependent on oxidative stress [37]. According to the results of bioinformatic prediction, five miRNA targets, preferentially involved in oxidative stress, as reported above, were identified for circADAM10 (mmu-miR-670-3p, mmu-miR-509-5p, mmu-miR-1903, mmu-miR-5106, mmu-miR-7220-3p) and circPCSK6 (mmu-miR-7033-5p, mmu-miR-667-5p, mmu-miR-191-3p, mmu-miR-3073a-3p; mmu-miR-6989-3p) (Figure S5A,B). 

### 2.6. Validation of DE-circRNAs in Mouse SPZ, Testis, and Epididymis

**Figure 4:**
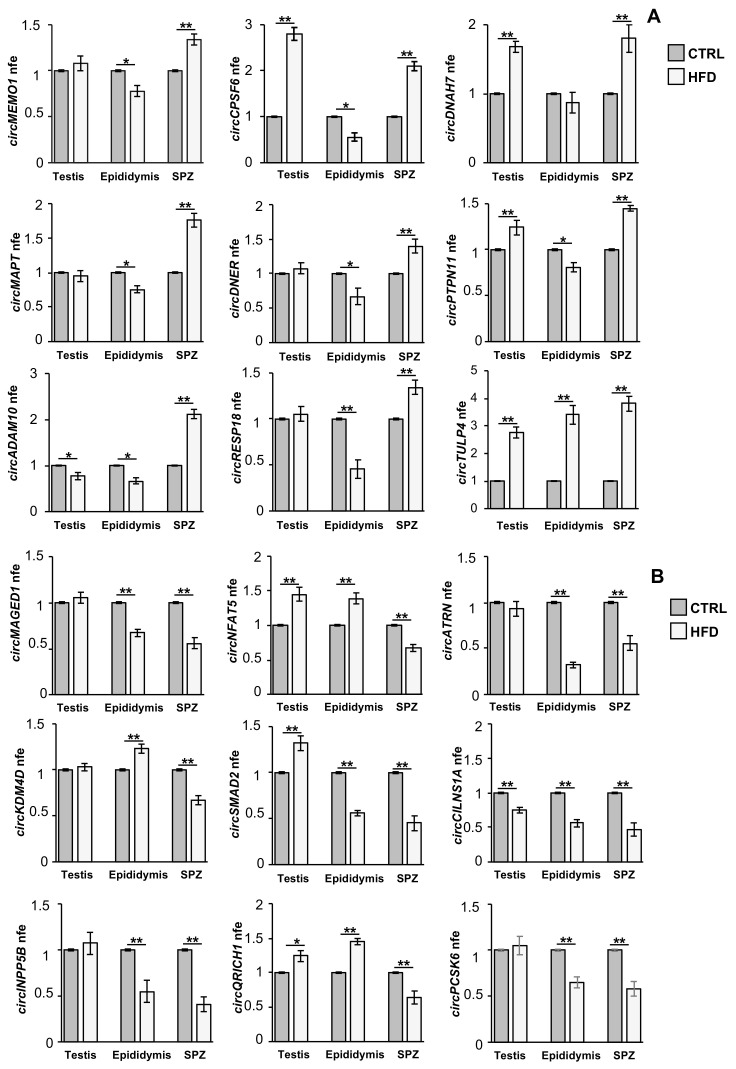
**Validation of circRNA microarray results in testis, epididymis, and SPZ from CTRL and HFD mice.** (**A**) Expression analysis of 9 circRNAs upregulated in HFD compared with CTRL-derived SPZ. (**B**) Expression analysis of 9 circRNAs downregulated in HFD compared with CTRL-derived SPZ. qRT-PCR data are normalized using *cyclophilin* and *ribosomal protein S18* (*RPS18*) for SPZ and tissues, respectively, expressed as fold expression (nfe) and reported as mean value ± S.E.M. **: *p* < 0.01; *: *p* < 0.05.

Selected DE-circRNAs, predicted to be involved in sperm physiology and characterized by a significant high score of normalized intensity, were chosen for circRNA microarray result validation by One-Step Evagreen qRT-PCR analysis. The relative sequences were searched in circBase (http://www.circbase.org, accessed on 9 March 2023) in order to design specific primer pairs for circular isoforms, spanning the backsplicing junction (Figure 4A,B). Accordingly, we checked the quality of melting curves for each primer pair in order to use for experimental validation only curves with single peaks. In all samples, to exclude blood and epididymal cell contaminations, we carried out the expression analysis of E-cadherin (epithelial cell marker) and CD4 (leukocyte marker). No significant signal was amplified by qPCR. In addition, as previously described, RNA extracted from SPZ was used as quality control [33]. 

The expression of circRNAs was evaluated in SPZ, as well as in testis and epididymis, in order to understand a putative testicular or epididymal derivation of spermatic circRNAs. The expression pattern of circRNAs upregulated (*p* < 0.01; Figure 4A) and downregulated (*p* < 0.01; Figure 4B) in HFD compared with CTRL SPZ, derived from qPCR analysis, confirmed circRNA microarray results. Interestingly, some circRNAs upregulated in HFD SPZ were overexpressed also in the testis, but not in the epididymis (circDNAH7, circPTPN11, circCPSF6, Figure 4A), suggesting a preferential testicular derivation, while others appeared upregulated only in SPZ (circADAM10, circRESP18, circMAPT, circDNER, Figure 4A) likely derived from an endogenous sperm backsplicing. On the other hand, several circRNAs downregulated in HFD SPZ showed a similar trend in the epididymis (circMAGED1, circATRN, circSMAD2, circINPP5B, circPCSK6, Figure 4B), suggesting an inefficient contribution of the epididymal epithelium in delivering circRNAs to SPZ, probably due to defects in epididymal circRNA biogenesis. 

### 2.7. FUS Orchestrates Spermatic and Epididymal Backsplicing

**Figure 5:**
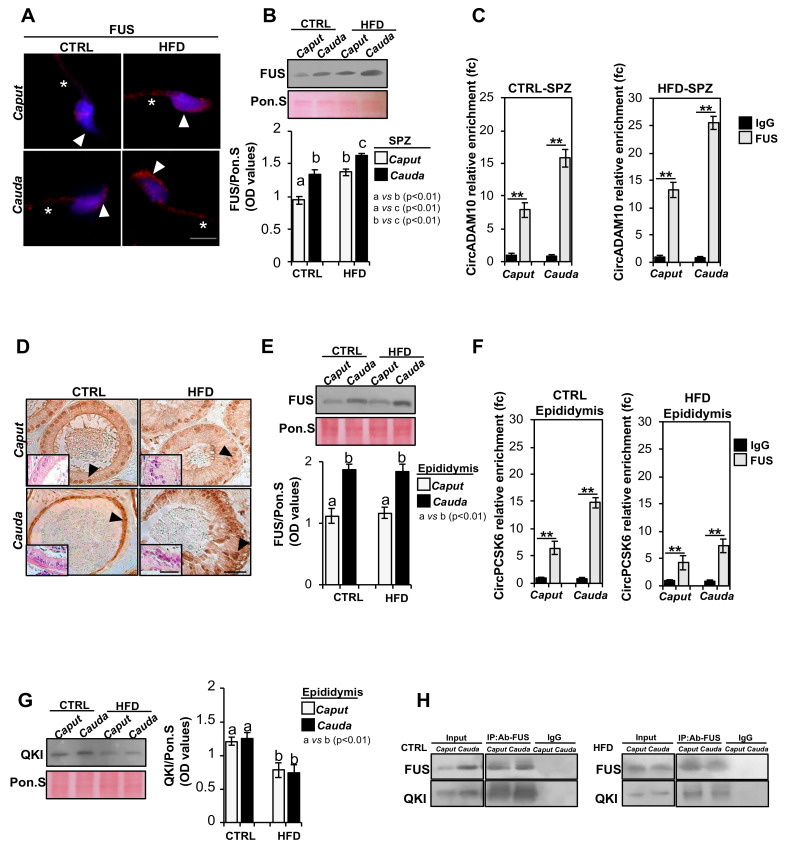
**Characterization of backsplicing machinery in SPZ and epididymis of CTRL and HFD mice.** (**A**) Immunofluorescence analysis of FUS protein in *caput* and *cauda* SPZ of CTRL and HFD mice. White arrowheads and white asterisks represent FUS localization (RED) in sperm head and tail, respectively. Nuclei were labeled with DAPI (blue). Scale bar: 5 µm. (**B**) Western blot analysis of FUS protein (75 KDa) in *caput* and *cauda* SPZ of CTRL and HFD mice (n = 5 different samples for each experimental group in triplicate). Signals were quantified by densitometry analysis and normalized to Ponceau Red (Pon.S). Data were expressed in OD values and reported as mean ± SEM. Experimental groups with statistically significant differences (*p* < 0.01) were indicated with different letters. (**C**) The enrichment levels of circADAM10 in the products of RIP assay (FUS-IP compared with IgG-IP) in *caput* and *cauda* SPZ of CTRL and HFD mice detected by qRT-PCR. Data are reported as mean  ±  SEM from three independent experiments. ** *p* < 0.01. (**D**) Immunocytochemistry of FUS in Bouin’s fixed *caput* and *cauda* epididymis sections (7 μm) of CTRL and HFD mice (n = 5 different samples for each experimental group in triplicate). The FUS protein localization in principal cells was indicated by black arrows. Scale bar: 50 μm; inset: H&E staining of Bouin’s fixed *caput* and *cauda* epididymis sections (7 μm) of CTRL and HFD mice; scale bar: 20 μm. (**E**) Western blot analysis of FUS protein in *caput* and *cauda* epididymis of CTRL and HFD mice. Signals were quantified by densitometry analysis and normalized to Ponceau Red (Pon.S). Data were expressed in OD values and reported as mean ± SEM. Experimental groups with statistically significant differences (*p* < 0.01) were indicated with different letters. (**F**) The enrichment levels of circPCSK6 in the products of RIP assay (FUS-IP compared with IgG-IP) in *caput* and *cauda* epididymis of CTRL and HFD mice detected by qRT-PCR. Data are reported as mean ± SEM from three independent experiments. ** *p* < 0.01. (**G**) Western blot analysis of QKI protein (40 KDa) in *caput* and *cauda* epididymis of CTRL and HFD mice. Signals were quantified by densitometry analysis and normalized to Ponceau Red (Pon.S). Data were expressed in OD values and reported as mean ± SEM. Experimental groups with statistically significant differences (*p* < 0.01) were indicated with different letters. (**H**) IP in *caput* and *cauda* epididymis of CTRL and HFD mice. Total proteins collected were immunoprecipitated using FUS antibody. Protein interaction between FUS and QKI was detected by Western blot analysis.

In order to clarify the molecular mechanisms underlying the DE-circRNA profile observed in HFD SPZ, we set two well-defined experimental strategies. First, we characterized the spermatic molecular profile of FUS, the main RBP orchestrating the backsplicing mechanism [29], to assess if DE-circRNAs upregulated in HFD SPZ were due to an enhanced FUS activity and/or content. SPZ collected from the *caput* and *cauda* epididymis of both CTRL and HFD mice were used for FUS immunofluorescence analysis (Figure 5A). As reported, FUS localization in *caput* CTRL SPZ showed a weak signal, detectable in both the sperm head and tail. According to our previous data [29], a more intense FUS signal, defined in the periacrosomal region and sperm tail, was detected in *cauda* SPZ of CTRL mice (Figure 5A). Interestingly, *caput* HFD SPZ early acquired the typical FUS localization and intensity observed in *cauda* CTRL SPZ, which progressively increased in *cauda* (Figure 5A). FUS protein levels were investigated in *caput* and *cauda* SPZ of CTRL and HFD mice by Western blot analysis (Figure 5B). FUS protein levels were higher (*p* < 0.01) in *cauda* than in *caput* CTRL SPZ; the same significant increase in FUS protein (*p* < 0.01) from *caput* to *cauda* was observed in HFD SPZ, but more interestingly, a higher FUS content was observed in both *caput* and *cauda* HFD SPZ when compared with FUS levels of CTRL SPZ ones, suggesting that HFD SPZ possessed an enhanced backsplicing activity (Figure 5B). To confirm this hypothesis, we carried out an RNA-binding protein immunoprecipitation (RIP) assay in *caput* and *cauda* SPZ of CTRL and HFD mice, using FUS antibody, to evaluate whether the increase in FUS observed in HFD SPZ was responsible for the increased backsplicing activity as to induce upregulation of DE-circRNAs. To achieve our goal, circADAM10 was chosen as representative circRNA upregulated in HFD SPZ. The results showed a significant 7.87- and 15.76-fold enrichment of circADAM10 (*p* < 0.01), relative to the use of IgG control, when the anti-FUS antibody was used in *caput* and *cauda* CTRL SPZ, respectively (Figure 5C). Similarly, a significant 13.24- and 25.49-fold enrichment of circADAM10 (*p* < 0.01), greater than observed in CTRL SPZ, was respectively observed in *caput* and *cauda* HFD SPZ (Figure 5C), confirming that HFD SPZ possessed enhanced endogenous backsplicing skills. 

Considering that circRNAs, downregulated in HFD SPZ, were likely dependent on defective epididymal circRNAs’ biogenesis, we performed a fine morphological and molecular characterization of FUS in CTRL and HFD epididymal sections, with the aim of investigating putative defects in epididymal backsplicing machinery. Immunocytochemistry analysis performed in *caput* and *cauda* epididymal sections of CTRL and HFD mice, using FUS antibody, showed a well-defined FUS localization in epididymal principal cells that progressively increases from *caput* to *cauda* in both CTRL and HFD epididymis (Figure 5D). Interestingly, HFD mice showed an anomalous epididymal morphology characterized by a severe disorganization in the layered epithelium of both *caput* and *cauda* epididymal segments, as shown by H&E staining (Figure 5D insets). Accordingly, Western blot analysis of the FUS protein carried out on *caput* and *cauda* epididymal lysates derived from CTRL and HFD mice showed an equal increase in FUS content (*p* < 0.01), from *caput* to *cauda*, in both experimental groups (Figure 5E), suggesting that epididymal backsplicing defects in HFD mice were not directly dependent on FUS protein content. Despite these data, we decided to carry out in the *caput* and *cauda* epididymis of CTRL and HFD mice, a RIP assay by using FUS antibody, to evaluate whether a defective backsplicing could still occur in the HFD epididymis. CircPCSK6 was chosen as representative circRNA downregulated in HFD epididymis. 

As reported, a significant 6.51- and 14.76-fold enrichment of circPCSK6 (*p* < 0.01), relative to the use of IgG control, was observed when the anti-FUS antibody was used in *caput* and *cauda* CTRL epididymis, respectively (Figure 5F). Similarly, a significant 4.23- and 7.32-fold enrichment of circPCSK6 (*p* < 0.01) was respectively observed in *caput* and *cauda* HFD epididymis (Figure 5F), nevertheless suggesting that backsplicing was less efficient in HFD than CTRL epididymis. 

Considering that FUS physically interacts with QKI to perform a successful backsplicing reaction [29], we investigated the QKI protein content in total lysates of *caput* and *cauda* epididymis derived from CTRL and HFD mice by Western blot analysis. As reported, QKI levels were constant from *caput* to *cauda* epididymis in both experimental groups; however, a significant reduction of QKI in *caput* and *cauda* HFD epididymis (*p* < 0.01) was observed when compared with CTRL one (Figure 5G). 

Then, we immunoprecipitated FUS from total proteins collected from *caput* and *cauda* of CTRL and HFD epididymis (IP-US), followed by immunoblotting with FUS and QKI antibodies (Figure 5H), in order to evaluate an inefficient FUS–QKI protein interaction. Immunoblotting with FUS and QKI antibodies showed both FUS and QKI signals in CTRL IP-FUS as compared with input signals, confirming the formation of heterodimeric complex in *caput* and *cauda* CTRL epididymis. Interestingly, IP-FUS carried out in total protein lysates of *caput* and *cauda* HFD epididymis showed a strong reduction of QKI signal, which appeared to be dependent on the reduction of QKI protein content, as confirmed by input samples’ (total lysates isolated before the immunoprecipitations) analysis (Figure 5H). These data suggested that an impaired backsplicing mechanism, dependent on an inefficient FUS–QKI protein complex formation, occurred in HFD epididymis, thus affecting epididymal-derived circRNA biogenesis. 

### 2.8. DE-circRNA Recovery in HFD SPZ Improves Sperm Motility 

In order to assess the involvement of DE-circRNAs in the regulation of sperm motility, we chose one circRNA downregulated in HFD SPZ and carried out the construction of a ceRNET in order to shed light on predicted mRNAs potentially regulating sperm motility pathways. We chose circSMAD2 since its linear transcript encodes for a protein involved in testosterone production as well as germ cell proliferation and apoptosis inhibition [38]. In addition, SMAD2 was reported to be mainly present in the cytoplasm of germ cells, and changes in its expression levels were associated with decreased sperm production in rats [39]. 

**Figure 6:**
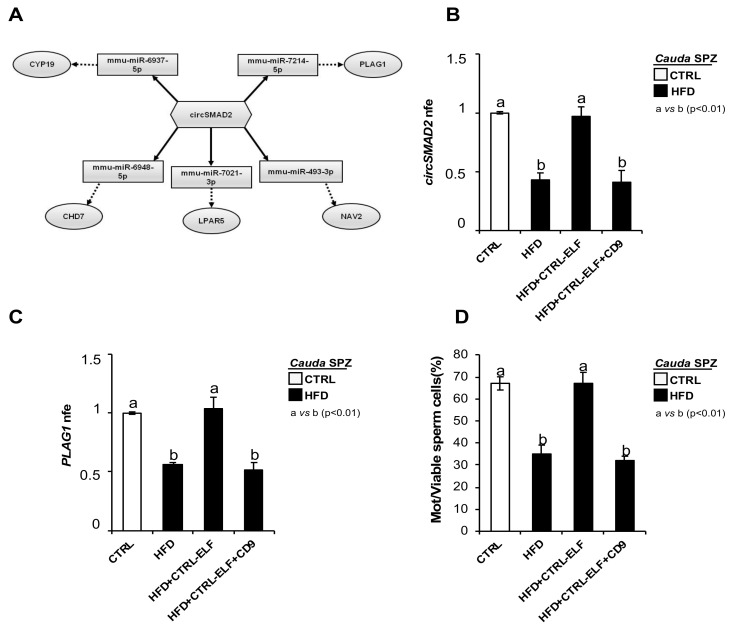
**In vitro experiment of vesicle shuttle: from CTRL epididymis to HFD SPZ.** (**A**) CeRNET of one circRNA downregulated in HFD SPZ. CircSMAD2 tethers a group of miRNAs as targets, all involved in sperm motility pathways. Networks were built using Cytoscape. Hexagonal and rectangular symbols represent circRNAs and miRNAs, respectively. The arrow indicates the tethering activity of circRNAs toward miRNAs, while the dotted arrow indicates the pathways upstream of the miRNAs. (**B**,**C**) qRT-PCR analysis of circSMAD2 (**B**) and *PLAG1*-mRNA (**C**) in CTRL *cauda* SPZ and HFD *cauda* SPZ in vitro coincubated with: PBS, CTRL-ELF, and CTRL-ELF pretreated with anti-CD9 antibody (n = 6 for each experimental group). qRT-PCR data were normalized using *cyclophilin*, expressed as fold expression (nfe) relative to CTRL SPZ and reported as mean value ± S.E.M. Experimental groups with statistically significant differences (*p* < 0.01) were indicated with different letters. (**D**) Sperm motility assay in CTRL *cauda* SPZ and HFD *cauda* SPZ in vitro coincubated with: PBS, CTRL-ELF, and CTRL-ELF pretreated with anti-CD9 antibody (n = 6 for each experimental group); data were expressed as the percentage of motile/live SPZ and reported as mean ± SEM. Experimental groups with statistically significant differences (*p* < 0.01) were indicated with different letters.

According to the results of bioinformatic prediction, five miRNA targets, preferentially involved in sperm motility were identified for circSMAD2 (mmu-miR-6737-5p, mmu-miR-7214-5p, mmu-miR-493-3p, mmu-miR-7021-3p, mmu-miR-6948-5p) (Figure 6A). Among the mRNA targets identified, more of them were described as key regulators of sperm motility as: (i) the reduction of CYP19, both transcript and enzymatic activity, has been correlated with sperm motility reduction [40]; (ii) lysophosphatidic acid (LPA) via LPA receptors (LPAR) improves sperm motility by enhancing the glycolysis pathway [41]; (iii) CHD7 gene missense variants occur in the azoospermia condition [42]; (iiii) PLAG1 deficiency negatively affects sperm motility in mice [43]; voltage-gated sodium channels NAV were identified in bull SPZ [44]. 

Considering that we have previously demonstrated that circRNAs downregulated in HFD SPZ were due to an inefficient epididymal backsplicing, and in turn dependent on an affected epididymal RNA molecule delivery to SPZ, we carried out an in vitro experiment of vesicle shuttle in order to demonstrate the contribution of circSMAD2, and of its related ceRNET, to HFD sperm motility recovery. In detail, HFD SPZ were collected from *cauda* epididymis, as this represents the spermatic population with the highest maturation grade of motility parameters, and they were incubated in PBS, CTRL-epididymal luminal fluid (ELF), and CTRL-ELF pretreated with CD9 antibody, respectively. CircSMAD2 levels were analyzed by qRT-PCR analysis in all experimental groups in comparison with *cauda* CTRL SPZ (Figure 6B). As reported, a significant increase in circSMAD2 to CTRL values (*p* < 0.01) was observed when HFD SPZ were incubated with CTRL-ELF (Figure 6B). This effect was counteracted by an anti-CD9 masking antibody approach, previously suggested to reduce the efficacy of RNA molecule transfer from epididymosomes to SPZ [29], confirming that the observed increase in circSMAD2 was exclusively dependent on CTRL-ELF vesicle uptake (Figure 6B). 

In order to validate circSMAD2-related ceRNET, *PLAG1* levels, chosen as representative mRNA target involved in sperm motility pathways, were investigated by qRT-PCR analysis (Figure 6C). As expected, *PLAG1* levels were significantly increased (*p* < 0.01) in HFD SPZ incubated with CTRL-ELF, and this effect was counteracted by an anti-CD9 masking antibody approach (Figure 6C). 

Finally, sperm motility was assessed to investigate the potential effects of this ceRNET restoring spermatic functional parameters (Figure 6D). Results showed a significant increase in CTRL values (*p* < 0.01) in the percentage of motile HFD SPZ when treated with CTRL-ELF. Accordingly, this effect was counteracted by an anti-CD9 masking antibody approach (Figure 6D), confirming that spermatic circSMAD2 recovery in *cauda* HFD SPZ improved sperm motility parameters. 

## 3. Discussion 

In developed countries, the marked increase in overweight and obesity, tightly related to lifestyle behaviors and sociocultural factors, is becoming an increasingly widespread and alarming phenomenon worldwide. Obesity-related dysfunctions not only heighten metabolic diseases’ onset, but have also been correlated with a severe decline in reproductive skills, leading to male infertility and assisted reproduction techniques’ (ART) failure [8,9,45,46,47]. 

CircRNAs, a new class of ncRNAs, are increasingly acquiring a prominent role in sperm quality setting; indeed, they have been identified in: (i) mammalian testis and seminal plasma [30,31] and (ii) human SPZ in both physiological and pathological conditions [33,34]. Spermatic circRNA cargo constitutes a novel “sperm RNA code” useful for assessing sperm quality, affecting embryo development as a consequence. Therefore, a topic of high relevance, consisting in the identification of a metabolically influenced spermatic circRNA cargo, has been assessed in the current work. 

To achieve our goal, male mice fed with HFD were used as experimental model. HFD phenotype validation clearly showed the typical biochemical and morphological features derived from the obesity condition as (i) increased body weight and abdominal circumference; (ii) liver steatosis; (iii) heart hypertrophy; and (iv) hyperlipidemic, hyperinsulinemic and hyperleptinemic phenotype. Interestingly, several anomalies were also observed for the reproductive functions. First, tubular seminiferous epithelium appeared severely compromised given the high germ cell detachment observed. Consistent with our results, Fan and colleagues demonstrated a direct correlation between obesity and blood–testis-barrier integrity decline, dependent on the affected expression of tight junction proteins as occludin and ZO-1 [48]; therefore, such a similar HFD-dependent effect in our experimental model appears plausible. 

Then, we examined the main sperm parameters fundamental for a successful fertilization process. Interestingly, HFD impaired sperm motility, as well as viability and head morphology. Accordingly, a correlation between testicular oxidative stress and/or inflammation and sperm head morphology has been reported [11] as well as sperm structural anomalies in various HFD-fed mice models [49,50]. 

Considering the growing attention given to deregulated ncRNAs in metabolic diseases and obesity onset [17,19,20,21] and the recent pieces of evidence concerning the paternally dependent effects on offspring health [51,52], here we decided to shed light on HFD effects on spermatic circRNA content, highlighting their putative role as modulators of oxidative stress and potential markers of paternal metabolic disorder inheritance. 

We profiled the circRNA expression pattern in CTRL and HFD epididymal SPZ by using a microarray experimental strategy. A total of 109 DE-circRNAs, consisting of 43 upregulated and 66 downregulated circRNAs, were identified in HFD compared with CTRL SPZ. Chromosomal analysis highlighted any DE-circRNA derived from host genes located on chromosomes chr20, chr21, chr22, chrY, and chrM. In addition, up- and downregulated DE-circRNAs identified in HFD SPZ showed the same tethering activity scheme, consisting of different DE-circRNAs able to sponge the same miRNA target. As highlighted by KEGG pathway analysis, DE-circRNAs in HFD SPZ appeared to be involved in biological pathways underlying metabolic processes as: (i) regulation of lipolysis in adipocytes; (ii) ovarian steroidogenesis; (iii) thermogenesis; and (iv) thyroid hormone synthesis. 

Then, we focused our attention on the predicted involvement of HFD DE-circRNAs in modulating the oxidative stress pathway. With this in mind, we pointed to circADAM10 and circPCSK6, up- and downregulated in HFD SPZ, respectively, to evaluate their regulatory ceRNETs, considering that the linear counterparts of both encode for key regulators involved in the prevention of the oxidative stress condition. Indeed, the pharmacological inhibition of ADAM10 leads to neuroinflammation and oxidative stress [36], while PCSK6 knockdown in cardiomyocytes increases ROS level and apoptosis in vitro [37]. 

CircADAM10—through mmu-miR-670-3p, mmu-miR-509-5p, mmu-miR-1903, mmu-miR-5106, and mmu-miR-7220-3p—controls the expression of several targets as (i) FAS-ligand, involved in enterocyte apoptosis following exposure to oxidative stress [53]; (ii) pyruvate kinase M2 isoform (PKM2), essential for ROS adaptation of cancer cells [54]; and (iii) nitric oxide synthase 3 (NOS3), implicated in testicular oxidative stress and oligoasthenozoospermia [55]. On the other hand, circPCSK6—through mmu-miR-7033-5p, mmu-miR-667-5p, mmu-miR-191-3p, mmu-miR-3073a-3p, and mmu-miR-6989-3p—controls the expression of: (i) MDM2, which acts on respiratory complex I activity enhancing mitochondrial ROS production [56]; (ii) HIF-1α, involved in the prevention of oxidative stress-induced apoptosis [57]; and (iii) the ROS-induced transcription factors NFR1, which regulates ROS signaling underpinning cancer cell survival [58]. 

The experimental validation of DE-circRNAs highlighted that two intriguing behaviors occurred in HFD SPZ and epididymis, respectively. In detail, several circRNAs, upregulated in HFD SPZ, appeared to be derived from enhanced sperm endogenous backsplicing activity, while most circRNAs, downregulated in HFD SPZ, likely seemed to be dependent on inefficient epididymal circRNA biogenesis, and thus on impaired epididymal circRNA delivery to SPZ. 

Previously, we identified FUS as the principal RBP able to orchestrate the endogenous formation of spermatic circRNA, and QKI as one of the favorite FUS molecular interactors needed for its RNA circulating skills [29,35]. Based on these pieces of evidence, we have morphologically and molecularly characterized FUS protein in both HFD sperm and epididymis in order to demonstrate its potential involvement in molecular mechanisms underlying both spermatic and epididymal circRNA biogenesis. In agreement with our previous results, the spermatic FUS content increased along the epididymal duct due to the well-characterized epididymal contribution via epididymosomes [29]. Surprisingly, FUS protein not only appeared quantitatively increased in HFD SPZ, but very early showed a cell redistribution typically acquired during epididymal transit, suggesting a greater activity of endogenous backsplicing machinery already in *caput* epididymis. The increased FUS-dependent circADAM10 biogenesis, both in *caput* and in *cauda*, confirmed our hypothesis. 

Based on these exciting results, we hypothesized that the production of circRNAs, downregulated in HFD SPZ, was dependent, unlike that demonstrated in isolated SPZ, on a defective epididymal backsplicing mechanism due to FUS downregulation. Thus, similar to what was performed in isolated SPZ, we carried out the molecular and morphological characterization of FUS in HFD epididymis. Unlike our hypothesis, the HFD epididymis showed for FUS similar levels of expression and localization in epididymal principal cells as CTRL one. Nevertheless, a strong deregulation of the epididymal epithelial architecture, characterized by the loss of the typical multilayered columnar arrangement of principal cells, was also observed in the HFD epididymis. Considering that epididymal layered epithelium is dependent on cell-to-cell junctions and is able to coordinate protein-to-protein interactions to ensure several biological processes [59,60,61,62,63,64], we asked if an anomalous backsplicing mechanism, dependent on impaired FUS molecular partner interactions, could occur in HFD epididymis. With this in mind, we pointed our attention to QKI, the main FUS partner recently reported to be involved in spermatic backsplicing endogenous skills [29,35]. Effectively, a drastic QKI reduction was observed in HFD epididymis, and in agreement, a strong reduction of FUS–QKI physical interaction occurred. Is the impairment of the FUS–QKI complex able to affect epididymal circRNA biogenesis? Once again, FUS-RIP experiments gave us the answer. Indeed, despite normal epididymal FUS levels, an inefficient FUS-dependent circPCSK6 backsplicing was observed in HFD epididymis, probably due to a poor FUS circularizing activity dependent on the loss of its key molecular partner, QKI. 

Lastly, we demonstrated the direct role of DE-circRNAs in modulating sperm motility. To achieve our goal, we set a well-defined experimental strategy. First, we built a ceRNET potentially involved in sperm motility modulation, focusing on circSMAD2, downregulated in HFD SPZ due to an inefficient epididymal contribution as reported above, and on *PLAG1*, its downstream target, as it encodes for a zinc finger transcription factor involved in sperm motility. Accordingly, PLAG1 knock-out mice showed a severe motility reduction in *cauda* SPZ [43]. Thus, we carried out an in vitro experiment of vesicle shuttle incubating *cauda* HFD SPZ with *cauda* CTRL-ELF in order to favor circSMAD2 and *PLAG1* uptake. As expected, HFD sperm motility was enhanced following CTRL epididymal RNA molecule delivery to SPZ, thus demonstrating how the recovery of a ceRNET favors normal sperm motility in HFD SPZ. 

In conclusion, the current work confirms HFD-dependent effects on functional and morphological sperm parameters and sheds light on how HFD may influence spermatic circRNA cargo, with the identification of paternal ceRNET potentially involved in sperm oxidative stress and motility anomalies. In addition, a dysregulation in backsplicing machinery was demonstrated in both HFD SPZ and epididymis.

## 4. Material and Methods

### 4.1. Experimental Animals 

This study was performed by using *Mus musculus* C57BL6/J mice (ENVIGO Srl, Udine, Italy). Eight-week-old male mice were arranged so that everyone occupied a single cage and randomly received a normal-fat diet (CTRL, n = 12) (Teklad Standard TD.2918; carbohydrate: 58%, protein: 24%, fat: 18% Kcals, ENVIGO) or high-fat diet (HFD, n = 12) (Teklad Custom Research Diet TD. 06414; carbohydrate: 21%, protein: 18%, fat: 60% Kcals, ENVIGO), for 12 weeks. 

Considering that the diet initiation age (3, 6, or 9 weeks of age) has a negligible impact on the final body weight or percent of body fat at 19 weeks [65], we chose to start the experimental feeding at 8 weeks and up to 20 weeks of age to reproduce the increase in body weight and fat mass of an established model of diet-induced obesity.

The treatment was performed in service (ENVIGO) that weekly recorded body weight increases (data shown in Supplemental Figure S1) and food intake to verify the experimental approach.

Before the sacrifice, at 20 weeks of age, body weight and abdominal length and circumference were measured in all animals. Blood was collected for serum measurements, and then, the animals were sacrificed by cervical dislocation when completely sedated with 4% isoflurane (Iso-Vet, Piramal Healthcare, UK Limited) for 5 min in a Plexiglass chamber, after making sure of the lack of heartbeat and reflex active paw. The peripheral tissues (testes, liver, heart) were rapidly removed, weighed, and stored at −80 °C for molecular investigations and/or fixed in Bouin’s solution for morphological analyses. The epididymides were accurately removed and processed for the collection of SPZ (total or by caput and cauda epididymis, separately) and related epididymal fragments, depending on the experimental procedure, as described below. 

The number of animals needed for this study was deducted from a preliminary study that allowed for carrying out the statistical evaluations necessary to estimate the number of each experimental group and was established using the G*Power analysis (latest ver. 3.1.9.7; Dusseldorf, Germany; http://www.gpower.hhu.de/; accessed on 2 July 2020). In detail, such an analysis suggested at least 3 animals/group to have 0.96 as power calculation, the *p*-value being fixed to 0.05. To have a useful power calculation (i.e., actual power of 0.96), we used the following parameters: 3.48 effect size, 0.05 (error probability), 0.8 power (1- b err prob). The use of these parameters returned that each group should be formed by at least 3 separate animals to have 0.96 as power calculation. Therefore, 3, 5, or 6 animals/experimental group were analyzed and found to be statistically significant. 

Experiments were approved by the Italian Ministry of Education and the Italian Ministry of Health, with authorization (no. 405/2021-PR). Procedures involving animal care were carried out in accordance with the National Research Council’s publication Guide for Care and Use of Laboratory Animals (National of Institutes of Health Guide).

### 4.2. Mouse Sperm Collection

Total epididymis (from n = 6 CTRL and n = 6 HFD) or *caput/cauda* epididymis (from n = 6 CTRL and n = 6 HFD) was separately immersed in PBS (pH 7.6) and cut into a few pieces to allow the sperm to flow out from the ducts. The sperm was eluted through cheesecloth to remove the tissue debris and centrifuged at 1500× *g* for 30 min at 4 °C. The pieces of total and *caput/cauda* epididymis, deprived of sperm cells, were separately frozen and/or fixed in Bouin’s solution. The sperm pellet was treated with somatic cell lysis buffer (SCLB; 0.1% SDS, 0.5% Triton X-100 in DEPC-H2O) for 30 min on ice to eliminate somatic cell contamination. The removal of somatic cells was checked by microscope evaluation. After lysis, SPZ were centrifuged at 800× *g* for 15 min at 4 °C and then washed twice with PBS. Some aliquots of total and *caput/cauda* epididymal SPZ were stored at −80 °C and used for molecular investigations, while others were dried on slides and stored at −20 °C for morphological analyses. In addition, aliquots of *cauda* SPZ were used for sperm functional analysis as above reported. 

### 4.3. Blood Measurements 

The blood samples of CTRL (n = 12) and HFD (n = 12) animals were collected at 12:00 p.m. and used to evaluate the serum levels of triglycerides, total cholesterol, HDL, LDL, insulin, and leptin and blood levels of glucose by a specialized screening service. Fasted values were collected after food removal at 6:00 a.m. Results were expressed as mean value ± SEM.

### 4.4. Mouse Sperm Functional Analysis

An aliquot of *cauda* SPZ (from n = 6 CTRL and n = 6 HFD) was used to analyze the sperm viability and motility. In brief, the number of live, motile, and total SPZ was evaluated by two observers under a light microscope (Leica CTR500, Leica Microsystems Inc., Milan, Italy) at a magnification of 20X using a hemocytometer (Burker Chamber). The procedure was validated by double-blind test. The live SPZ, assessed through the viable dye trypan blue reagent (trypan blue, 0.4% solution, 17-942E Lonza), were used to define the percentage of dead/total SPZ. The motile SPZ were expressed as percentage of motile/live SPZ. For both assays, a minimum of 100 sperm cells were evaluated and counted for each analysis. 

### 4.5. Total RNA Preparation 

Murine tissues (testis and epididymis) and SPZ collected from CTRL and HFD male mice were used to extract total RNA by using TRIzol Reagent (Invitrogen Life Technologies, Paisley, UK) following the manufacturer’s instructions. All samples were homogenized in an adequate volume of TRIzol Reagent (1 mL TRIzol Reagent/mg tissue or 5–10 × 10^6^ sperm cells) for 5 min at 20 °C. Following the addition of 0.2 mL chloroform/mL TRIzol Reagent, the samples were centrifuged at 12,000× *g* for 15 min at 4 °C in order to obtain an aqueous phase in which the addition of isopropyl alcohol (0.5 mL/mL TRIzol Reagent) and 1 µL glycogen (20 mg/mL) ensured total RNA precipitation. Then, samples were centrifuged at 12,000× *g* for 10 min at 4 °C to obtain the RNA pellet. Each RNA pellet was washed with 75% ethanol, centrifuged at 7500× *g* for 10 min at 4 °C, and dissolved in DEPC-treated water. A NanoDrop 2000 spectrophotometer (Thermo Fisher Scientific, Waltham, MA, USA) was used to quantify (ng/µL) the total RNA and to evaluate its purity (260/280 and 260/230 ratios). The treatment with 2U DNase I (RNase-free DNase I, Ambion, Thermo Fisher Scientific, Waltham, MA, USA) was carried out on RNA aliquots (10 µg) to remove genomic DNA contamination. The RNAs were then preserved at −80 °C until the next step. 

### 4.6. CircRNA Microarray 

The CircRNA profile was obtained by using microarray analysis (Arraystar, Rockville, MD, USA). According to the Arraystar Super RNA Labeling protocol (Arraystar, Inc.), a random priming method was applied to amplify and transcribe into fluorescent cRNA the enriched ciRNAs. Then, labeled cRNAs were purified using the RNeasy Mini Kit (Qiagen, Germantown, MD, USA). A fragmentation step was carried out by adding 5 μL of 10 × blocking agent and 1 μL of 25 × fragmentation buffer to 1 μg of each labeled cRNA, which was heated at 60 °C for 30 min. As a final step, labeled circRNAs were diluted with 25 μL of 2 × hybridization buffer.

CircRNA expression microarray slides were assembled, dispensing 50 μL of hybridization solution into the gasket slide, which were incubated for 17 h at 65 °C in an Agilent Hybridization Oven. The hybridized arrays were washed, fixed, and scanned using the Agilent Scanner G2505C (Agilent Technologies, Santa Clara, CA, USA). 

### 4.7. Profiling Data and Differential Expression Analysis 

Agilent Feature Extraction software (version 11.0.1.1) was used to acquire and analyze array images, and then, the R software package (version 4.2 Limma, Bioconductor) was used as a tool for raw data quantile normalization and data processing. Only the circRNAs where at least 3 out of 6 samples have flags in “P” or “M” (“All Targets Value”) were retained for further analyses. The R software package was used to select DE-circRNAs, with *p*-value < 0.05 and fold change > 1.5, from the two groups of circRNAs identified in CTRL and in HFD SPZ. We used along the text the expression “HFD compared to CTRL SPZ” to indicate statistically significant DE-circRNAs, as shown by volcano plot filtering. Finally, fold change filtering was used to identify DE-circRNAs among samples, while a hierarchical clustering was performed to show the distinguishable circRNA expression pattern among samples. 

### 4.8. Functional Annotation for circRNA/miRNA and Target miRNA Interaction

Arraystar’s miRNA target prediction was used as bioinformatic tool, based on TargetScan [66] and MiRanda online analytical software, version 1.0 [67], to predict the circRNA/miRNA interaction pathways for all DE-circRNAs. In addition, DAVID Bioinformatics Resources 6.8 (david.ncifcrf.gov/home.jsp, accessed on 8 February 2021) was used to selectively perform gene ontology (GO) enrichment and Kyoto Encyclopedia of Genes and Genomes (KEGG) analyses (www.genome.jp/kegg, accessed on 9 March 2023) on DE-circRNA host genes. A hypergeometric test corrected by Benjamini–Hochberg adjustment was used to calculate the *p*-value. Diana TarBase 8.0 (http://www.microrna.gr/tarbase, accessed on 9 March 2023) was used to predict miRNA targets, while the Bisogenet plug-in of Cytoscape (www.cytoscape.org, accessed on 9 March 2023) was used to build a circRNA/miRNA/target network (ceRNET). 

### 4.9. RNA Expression Analysis by One-Step Evagreen qRT-PCR

We investigated circRNA and *PLAG1* mRNA expression through a One-Step Evagreen qRT-PCR reaction kit containing a qRT-PCR enzyme mix, and an Evagreen qPCR Mastermix (Applied Biological Materials Inc., Ferndale, WA, USA) was used to carry out circRNA expression analysis on a CFX-96 Real Time PCR System (Bio-Rad, Milano, Italy). Assays were carried out in triplicate and included a negative control without RNA. In addition, melting curve analysis of primer pairs was conducted. CFX Manager software (Bio-Rad, Milano, Italy) was used for RNA expression analysis. *Cyclophilin* and *ribosomal RPS18* were chosen as housekeeping genes for SPZ and tissues, respectively, for data normalization. The normalized fold expression (nfe) of circRNAs was calculated by applying the 2^−∆∆Ct^ method. All the results were expressed as a mean value of nfe ± SEM. 

### 4.10. Histology and Immunohistochemistry Analysis

Murine tissues (testes, liver, heart, *caput/cauda* epididymis) (from n = 5 animals for each experimental group) collected from CTRL and HFD mice were fixed overnight in Bouin’s solution, embedded in paraffin and used for histological analysis. For H&E, microtome serial sections (7 μm thick) were cut and processed using standard procedures. For immunohistochemistry staining, *caput/cauda* epididymal tissue sections (7 μm thick) were deparaffinized, rehydrated, and permeabilized with PBS pH 7.4 containing 0.1% Triton X-100. The antigen retrieval step was realized in citrate buffer 0.01 M (pH 6.0), while the blocking was realized in PBS containing 5% BSA and normal goat serum (diluted 1:5). The sections were incubated overnight at 4 °C with anti-FUS (PA5-52610; Invitrogen, Milano, Italy) antibody (diluted 1:100). The avidin/biotin and the substrate/chromogen H_2_O_2_/DAB system were used to reveal the immunoreactivity. 

SPZ collected from *cauda* epididymis of CTRL and HFD mice (n = 6 for each experimental group), dried on slides, were processed for H&E staining using ordinary procedures. The tissue sections or sperm slides were observed under a light microscope (Leica CTR500, Leica Microsystems Inc., Milan, Italy) and photographed, and finally, the images were captured using a high-resolution digital camera (Leica DC300F). 

Additionally, CTRL and HFD heart sections were considered to calculate the diameter of the cardiomyocytes using a measurement module tool provided by IM1000 software version 4.0 (Leica Microsystems Inc., Wetzlar, Germany). A minimum of 4 random serial sections were evaluated, and the mean diameter, expressed in micron (µm), for each cardiomyocyte was calculated. Results were graphed as mean values of 3 serial sections ± SEM. To quantify the number of SPZ with anomalous head, a minimum of 100 sperm cells were considered and counted for each assay, reporting the data as the percentage of anomalous sperm head/total SPZ. All the results were validated twice by the same operator. 

### 4.11. Immunofluorescence Analysis 

Sperm cells collected from *caput/cauda* epididymis of CTRL (n = 4) and HFD (n = 4) mice, dried on slides as reported above, were used for immunofluorescence analysis. In detail, sperm cells were fixed with a fix solution consisting of 4% paraformaldehyde (sc-281692; Santa Cruz Biotechnology, Heidelberg, Germany) for 20 min at RT. Then, a permeabilization step was carried out by using 0.1% Triton X-100 (X100; Sigma-Aldrich, Milano, Italy). Blocking was conducted with 10% of donkey serum (ab7475; Abcam, Cambridge, UK) for 30 min at RT, and then sperm cells were incubated with anti-FUS antibody (PA5-52610; Invitrogen, Milano, Italy) overnight at 4 °C. A negative control consisting of primary antibody omission and an isotype control by using the same isotype (IgG) at the same concentration of FUS primary antibody (rabbit IgG, polyclonal isotype control (ab37415) from Abcam, Cambridge, UK) were carried out (Figure S6). Slides were washed 3 times in Dulbecco’s PBS (DPBS, 1X) until the addition of Texas Red conjugated antibody (Jackson ImmunoResearch, Cambridge, UK) for 1 h at 37 °C. Nuclei visualization was performed, incubating the slides with DAPI solution (D9542; Sigma-Aldrich, Milano, Italy). Immunofluorescence analysis was conducted under an optical microscope (Leica DM 5000 B + CTR 5000) with a UV lamp. 

### 4.12. Protein Extraction and Western Blot Analysis

Total protein extraction was performed for *caput/cauda* epididymal tissue, as well as SPZ collected from CTRL and HFD mice (n = 5 animals for each experimental group). The samples were processed in RIPA buffer (PBS, pH 7.4, 10 mM of dithiothreitol, 0.02% sodium azide, 0.1% SDS, 1% NP-40, and 0.5% sodium deoxycholate, in the presence of protease inhibitors (10 μg/mL of leupeptin, aprotinin, pepstatin A, chymostatin, and 5 μg/mL of TPCK)) to obtain total protein extracts. In detail, all samples were homogenized, sonicated 3 times for 30 s bursts, each at 60 mW, incubated on ice for 30 min, and centrifuged at a maximum speed of 30 min at 4 °C to collect supernatant consisting of total lysates. SDS-PAGE was used to separate total lysate and these latter were then transferred to polyvinylidene difluoride membrane (GE Healthcare, Milano, Italy) at 280 mA for 2.5 h at 4 °C. The blocking step (5% nonfat milk, 0.25% Tween 20 in Tris-buffered saline (TBS, pH 7.6)) was carried out for 3 h at RT. Then, the filters were incubated with different primary antibodies (anti-FUS (molecular weight of 75 KDa; PA5-52610; Invitrogen, Milano, Italy) and anti-QKI (molecular weight of 40 KDa; PA5-87292; Invitrogen, Milano, Italy)) dissolved in TBS-milk buffer (TBS pH 7.6, 3% nonfat milk) overnight at 4 °C. After washing in 0.25% Tween 20/TBS, filters were incubated with 1:1000 horseradish peroxidase-conjugated mouse IgG (Dako Corp., Milano, Italy) in TBS-milk buffer and then washed again. Finally, the enhanced chemiluminescence Western blotting detection system (Amersham ECL Western Blotting Detection Reagent (RPN2106), GE Healthcare, Milano, Italy) was used to detect the immune complexes. The specificity of the immunoreactions was routinely checked by omitting the primary antibody. 

### 4.13. Protein Immunoprecipitation (IP)

*Caput/cauda* epididymis and SPZ collected from CTRL and HFD male mice (n = 3 samples for each experimental group) were used for IP experiments. All samples were lysed in RIPA buffer to obtain total lysates as reported above. A concentration of 500 μg of supernatant proteins from each sample was incubated with 2 μg of anti-FUS primary antibody (PA5-52610; Invitrogen, Milano, Italy) or IgG (12370; Sigma-Aldrich, Milano, Italy), and placed under rotary agitation at 4 °C overnight. Each sample was then incubated for 4 h at 4 °C with an opportune volume of protein A/G PLUS agarose beads (sc-2003; Santa Cruz Biotechnology, Heidelberg, Germany). Then, samples were washed in 500 µL of cold TBS pH 7.6 3 times (3000× *g* for 3 min a 4 °C) and boiled in Laemmli sample buffer for 10 min to be later analyzed by SDS-PAGE. 

### 4.14. RNA-Binding Protein Immunoprecipitation Assay (RIP)

*Caput/cauda* epididymis and SPZ collected from CTRL and HFD male mice (n = 3 samples for each experimental group) were used for RIP experiments. All samples were lysed in RIPA buffer to obtain total lysates as reported above. Then, 5 µg of FUS antibody (PA5-52610; Invitrogen, Milano, Italy) or IgG (12,370; Sigma-Aldrich, Milano, Italy) was incubated at 4 °C overnight under rotary agitation with an equal concentration of protein lysate. Each sample was then incubated for 4 h at 4 °C with an opportune volume (60 µL) of protein A/G PLUS agarose beads (sc-2003; Santa Cruz Biotechnology, Heidelberg, Germany). After washing with cold TBS pH 7.6, bead pellets were used to carry out total RNA extraction by using TRIzol Reagent (Invitrogen Life Technologies, Paisley, UK) as reported above. Finally, a NanoDrop 2000 spectrophotometer (Thermo Fisher Scientific, Waltham, MA, USA) was used to quantify the immunoprecipitated RNAs with FUS and control IgG. The immunoprecipitated RNAs were stored at −80 °C until circADAM10 and circPCSK6 qRT-PCR analysis. 

### 4.15. Vesicle Shuttle In Vitro Experiment 

*Cauda* epididymis collected from CTRL and HFD mice (n = 6 samples for each experimental group) were separately immersed in PBS (pH 7.6) and cut to let SPZ flow out from the ducts. Then, SPZ samples were filtered through cheesecloth to eliminate epididymal tissue fragments and centrifuged at 1500× *g* for 30 min at 4 °C. The supernatant yielding the ELF was clarified via centrifugation (16,000× *g* for 30 min at 4 °C) as previously reported [29]. HFD SPZ pellets and ELF derived from CTRL epididymis were used for in vitro incubation as reported. In detail, 10 × 10^6^ SPZ from HFD *cauda* epididymis were incubated for 30 min at 37 °C in 1 mL of: (1) PBS (pH 7.6), (2) CTRL ELF, (3) CTRL ELF pre-treated for 2 h at 37 °C with anti-CD9 antibody (sc-13118; Santa Cruz Biotechnologies, Heidelberg, Germany) at concentration of 10–1000 ng/mL [29]. After treatment, SPZ were used for motility assay and then centrifuged at 1500× *g* for 20 min at 4 °C, washed in PBS, and stored at −80 °C for molecular investigations. 

### 4.16. PCR Primer Design

The primers to validate and amplify circRNAs of interest in murine SPZ were designed by the online tool Primer-BLAST (http://www.ncbi.nlm.nih.gov/tools/primer-blast/, accessed on 15 February 2021). In particular, we designed primers spanning the backsplicing junction, specific for the circular isoforms. In addition, cyclophilin and specific primers were designed and used for normalization in SPZ and tissues, respectively. Primer sequences are shown in Table 3. 

### 4.17. Statistical Analysis 

Statistical significance between groups was analyzed by one-way analysis of variance (ANOVA), followed by Student’s *t*-test and Duncan’s test (for multigroup comparison). Data were expressed as the mean± SEM of at least 5 independent animals for each experimental group. For qRT-PCR and Western blot analyses, triplicates from each of 5 animals/experimental group were considered. *p* < 0.05 and 0.01 were considered statistically significant.

### 4.18. Limitations of the Study

The current study has some limitations. The major limitation is the sample size used for discovery and validation. Furthermore, studies on mice could be biased with respect to experimental design because confounding factors, such as the stress for laboratory conditions, could affect the variability of results and even compromise animal welfare. Thus, a variability related to the housing environment and husbandry practices or possible aversion and/or anxiety-like behavior should be considered. 

Finally, as a preliminary approach, only few circRNAs were validated, and some regulatory ceRNETs were deepened. Starting from the pieces of evidence highlighted here, a wider cohort study needs to be evaluated in further studies.

### 4.19. A Schematic View of the Experimental Design

**Figure 7:**
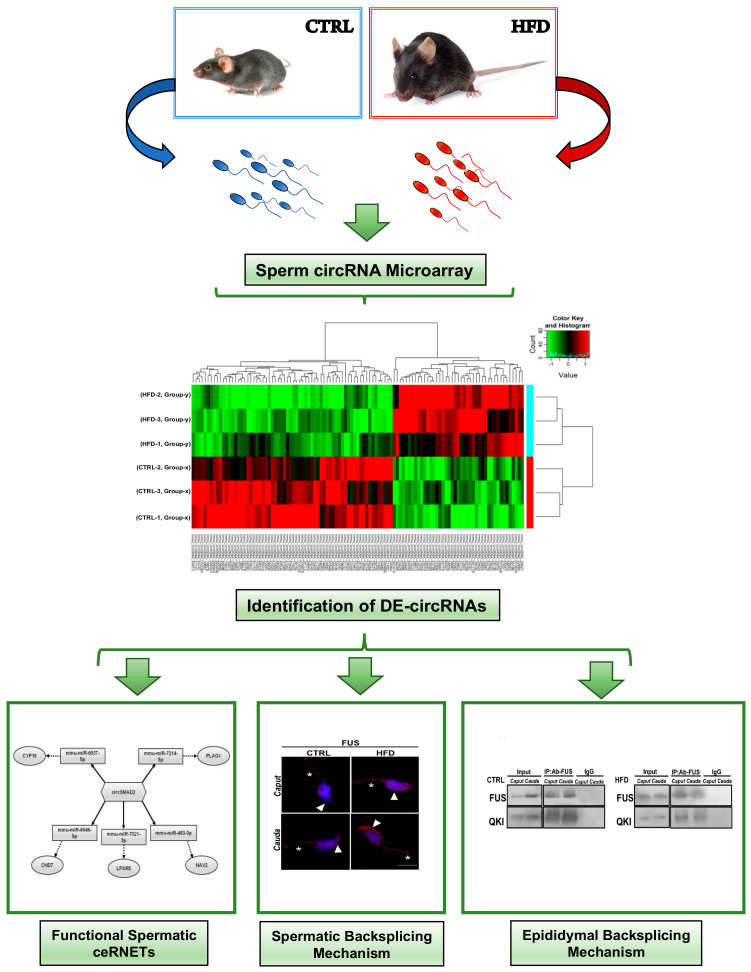
Schematic representation of experimental design.

A detailed representation of the whole experimental design is shown in Figure 7.

## 5. Conclusions

Obese men show reproductive dysfunctions. The sperm quality in these people needs to be checked, and a sperm RNA code is a useful instrument to assess it in order to prevent embryo development anomalies. In this frame, circRNAs are powerful molecules to investigate. By using an HFD-fed mice model, we discovered in SPZ a cargo of circRNAs differentially expressed in comparison with CTRL animals, involved in the oxidative stress pathway and sperm motility modulation. More interestingly, SPZ collected from HFD mice display an enhanced circRNA biogenesis, while their epididymis inefficiently works in circRNA biogenesis. FUS and its molecular interactor, QKI, orchestrate this mechanism.

## Figures and Tables

**Table 1:** Effects of HFD on body components. Data are expressed relative to the CTRL group and reported as mean ± SEM; * *p* < 0.05; ** *p* < 0.01.

	Liver (g)	Heart (g)	DX Testis (g)	SX Testis (g)
**CTRL**	1.28 ± 0.03	0.20 ± 0.02	0.09 ± 0.02	0.08 ± 0.01
**HFD**	1.57 ** ± 0.02	0.31 ± 0.04 *	0.08 ± 0.01	0.09 ± 0.02

**Table 2:** Blood levels of glucose, lipids, and hormones related to HFD phenotype. Data are expressed relative to the CTRL group and reported as mean ± SEM; * *p* < 0.05; ** *p* < 0.01.

	CTRL	HFD
**Triglycerides (mmol/L)**	0.95 ± 0.09	12.11 ± 0.08 **
**Total Cholesterol (mmol/L)**	1.91 ± 0.15	4.54 ± 0.19 **
**HDL^a^ (mmol/L)**	1.38 ± 0.12	4.67 ± 0.19 **
**LDL^b^ (mmol/L)**	0.67 ± 0.07	1.13 ± 0.05 *
**Fasted Glucose (mg/dl)**	115 ± 3.02	146 ± 4.91 *
**Fasted Insulin (ng/mL)**	0.9 ± 0.13	4.99 ± 0.59 **
**Non-Fasted Leptin (ng/mL)**	5.5 ± 0.72	84.8 ± 6.04 **

**^a^** High-density lipoprotein; **^b^** low-density lipoprotein.

**Table 3:** Primer sequences and annealing temperatures.

Gene Primers	Sequences 5′-3′	Tm (°C)
*Mmu circ-MEMO1* S	ACTATGATGAATCCCAGGGGG	52
*Mmu circ-MEMO1* AS	CAGGGGCACATGATGGGAAG	
*Mmu circ-DNAH7* S	TACACGGGCCCTGCATTGTA	57
*Mmu circ-DNAH7* AS	AGGAGAGACCCAGCATGTGTA	
*Mmu circ-MAPT* S	GTCAGGTCGAAGATTGGCTCT	55
*Mmu circ-MAPT* AS	ATACTGGTTCAAAGCCTTGCC	
*Mmu circ-DNER* S	TGTGTCCTAGACCCATGCAG	53
*Mmu circ-DNER* AS	TCTGCAACAAACTTCCAGACAC	
*Mmu circ-CPSF6* S	TCGTTAGAAGATTTGCCCTTGT	52
*Mmu circ-CPSF6* AS	ACAACAGGACTCTGACCATGA	
*Mmu circ-PTPN11* S	TACGGGGTCATGCGTGTTAG	53
*Mmu circ-PTPN11* AS	GGGGTGAAACCATTTGTCCG	
*Mmu circ-ADAM10* S	CCTATGTCTTCACAGACCGGG	55
*Mmu circ-ADAM10* AS	TGGGGATAGTCTGAAGGTGC	
*Mmu circ-RESP18* S	TCTCCCCAAAAGATGGTCAGG	53
*Mmu circ-RESP18* AS	TGCCTTCGGGTACAATCTGG	
*Mmu circ-TULP4* S	ATAAACTTCAACCTGCGAGGC	53
*Mmu circ-TULP4* AS	TCCCGGTTAATTCAGGAGCCA	
*Mmu circ-MAGED1* S	TGGCTTCTTCTGGGTTGAGTAAAT	52
*Mmu circ-MAGED1* AS	AGTAAAGGCCTGGGGAAACCTG	
*Mmu circ-CLNS1A* S	ACTGTTGCCGGACAGTTTGA	53
*Mmu circ-CLNS1A* AS	CAGGACAGGCGGTGTATAGT	
*Mmu circ-INPP5B* S	CCTCCTGCCAGTGAGAAGTG	58
*Mmu circ-INPP5B* AS	TCAGCTGAGTGATGTTCTTTCCT	
*Mmu circ-SMAD2* S	TGACTACACCCACTCCATTCC	56
*Mmu circ-SMAD2* AS	TCAGAGCAAGTGCTGGGATGT	
*Mmu circ-KDM4D* S	AAACTTGGGGCTTGAAGCGT	54
*Mmu circ-KDM4D* AS	GGGGTGCAGCAGAATCTCTT	
*Mmu circ-NFAT5* S	AAAAGAGCACTCGTGCCAGA	52
*Mmu circ-NFAT5* AS	TCAGAGAATTGCATAAAATGGGG	
*Mmu circ-QRICH1* S	TGGGAATGTAAGCAGCTTGGG	56
*Mmu circ-QRICH* AS *Mmu circ-ATRN* S *Mmu circ-ATRN* AS *Mmu circ-PCSK6* S *Mmu circ-PCSK6* AS	TGACAATCCACCTGAGGCT GCGTATGACCTGACTTCTAGGG TGAGCATCCAGGACCTTATGT GGAGTGGACTCTGGAAGTGC ACCTTGGCGACCCTTGTTTC	56 58
*RPS18* S	GAGACTCTGGATGCTAACTAG	56
*RPS18* AS	GGACATCTAAGGGCATCACAG	
*Cyclophilin-A* S	TGGTCTTTGGGAAGGTGAAAG	52
*Cyclophilin-A* AS	TGTCCACAGTCGGAAATGGT	
*PLAG1* S	CCATACACATGACCCCAACA	58
*PLAG1* AS	CTTCACACCCCCAGATGACT	

## Data Availability

The datasets in this study are available from the corresponding author upon reasonable request.

## Supplementary Materials

The following supporting information can be downloaded at: https://www.mdpi.com/article/10.3390/ijms24076865/s1.

## Abbreviations

ANOVA: one-way analysis of variance; ART: assisted reproduction techniques; ceRNA: competitive endogenous RNA; ceRNET: competitive endogenous RNA network; CTRL: control; DE: differentially expressed; DPBS: Dulbecco’s PBS; ELF: epididymal luminal fluid; FC: fold change; FUS: fused protein in sarcoma; GO: gene ontology; H&E: hematoxylin and eosin; HDL: high-density lipoprotein; HFD: high-fat diet; IP: protein immunoprecipitation; KEGG: Kyoto Encyclopedia of Genes and Genomes; LDL: low-density lipoprotein; LPA: lysophosphatidic acid; LPAR: LPA receptors; ncRNAs: noncoding RNAs; nfe: normalized fold expression; NOS3: nitric oxide synthase 3; PKM2: pyruvate kinase M2 isoform; QKI: quaking protein; RBPs: RNA-binding proteins; RIP: RNA-binding protein immunoprecipitation; RNApol2: RNA polymerase II; RPS18: ribosomal protein S18; SCLB: somatic cell lysis buffer; SPZ: spermatozoa.
