# Supplementary material for: Novel Insights into circRNA Saga Coming from Spermatozoa and Epididymis of HFD Mice
Source: Int J Mol Sci. 2023 Apr 6;24(7):6865. doi: 10.3390/ijms24076865 (PMC10095261; doi:10.3390/ijms24076865)
Supplement: Supplementary file 1 [file ijms-24-06865-s001.zip › ijms-2305176-supplementary.pdf]

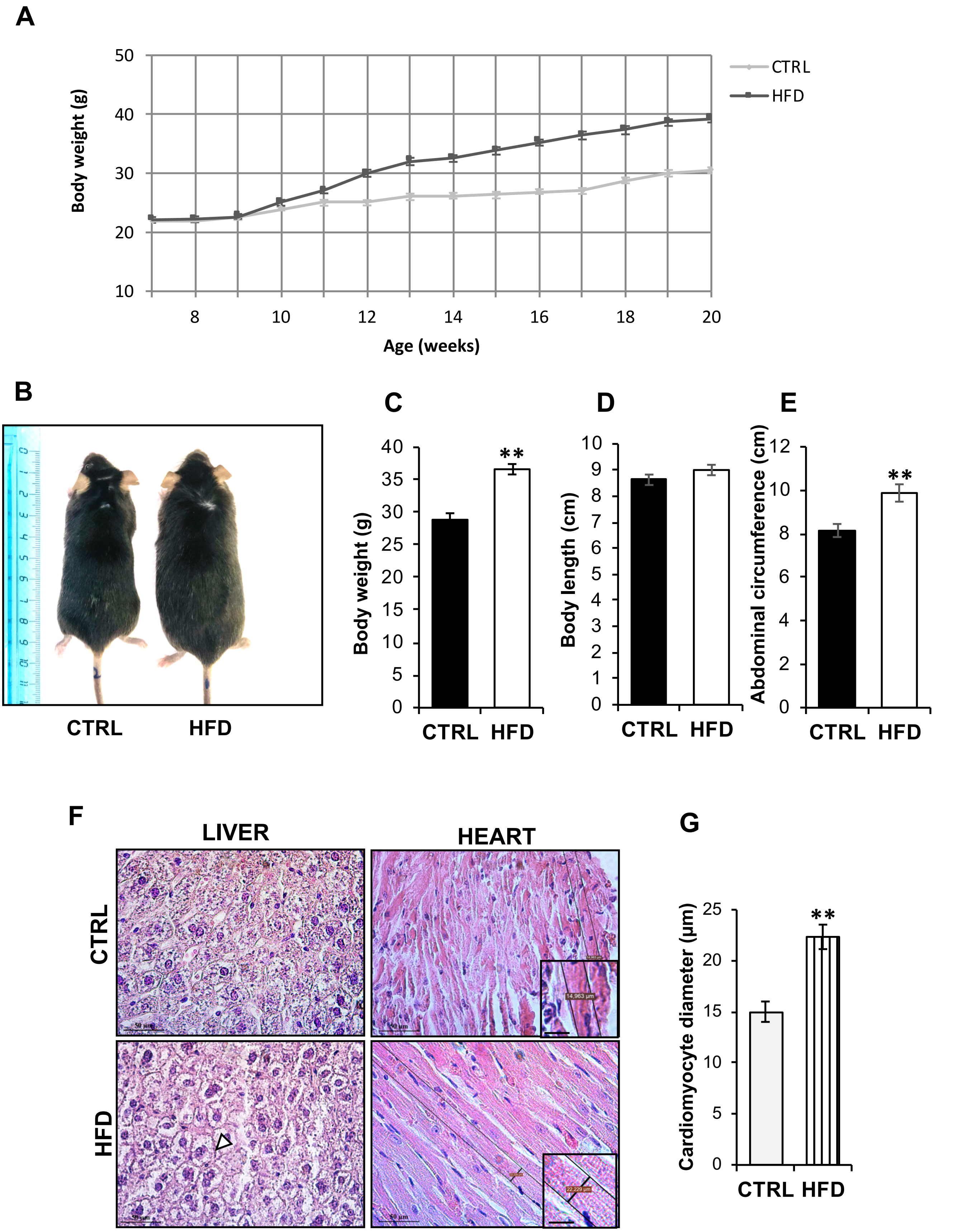

Figure S1. Description of HFD male mouse model.
(**A**) Body weights measurements of CTRL and HFD groups during the experimental protocol. (**B**) Representative image of CTRL and HFD male mice. (**C-E**) Body weight (**C**), body length (**D**) and abdominal circumference (**E**) in CTRL (n=12) and HFD (n=12) mice. (**F**) H&E staining of Bouin's fixed CTRL and HFD liver, and heart sections (7 µm). Fat vacuole accumulation in hepatic cell were indicated by white arrowheads; cardiomyocyte longitudinal areas were reported in the insets. Scale bar: 100 µm; scale bar inset: 20 µm. (**G**) Cardiomyocyte diameter measurements in CTRL and HFD mice. All data are reported as mean ± SEM; **p<0.01.

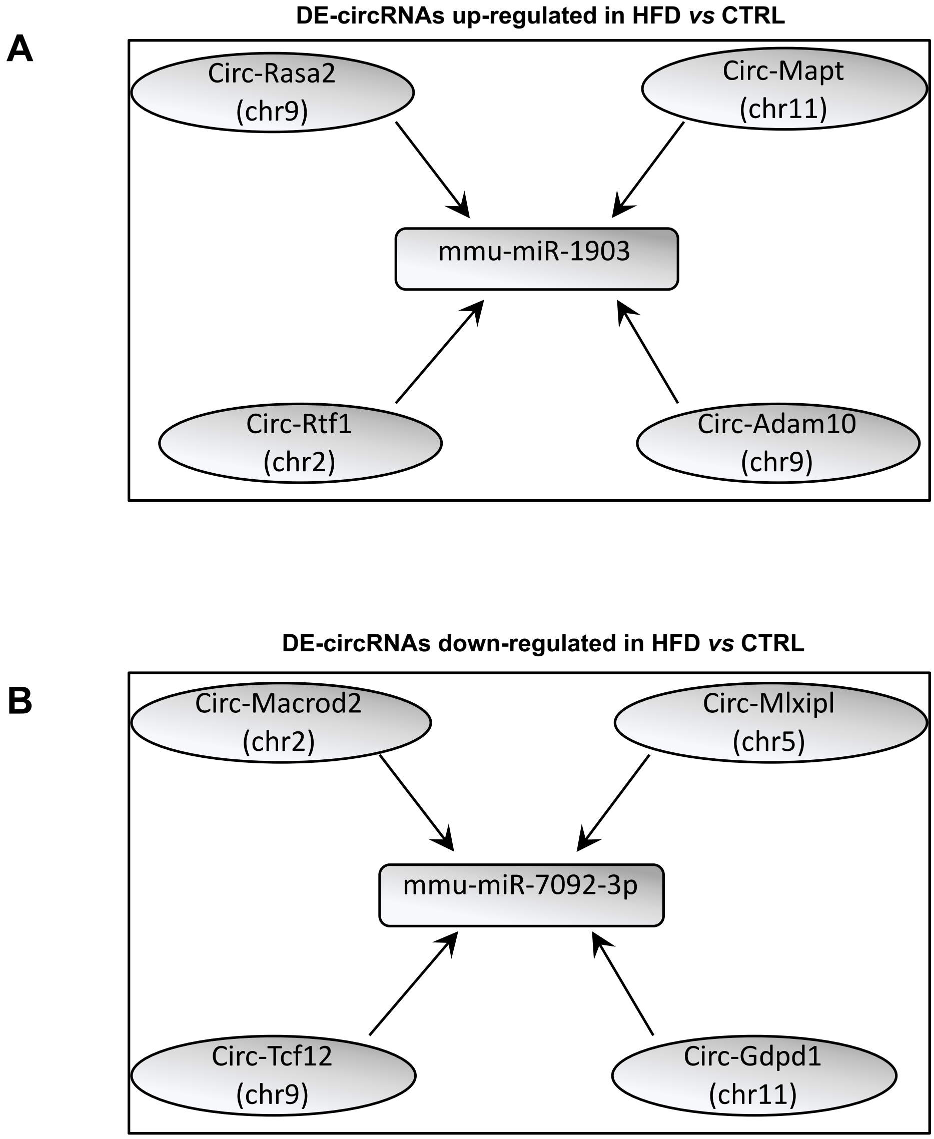

Figure S2. Functional clustering of DE-circRNAs in HFD compared to CTRL SPZ. **(A-B)** Four circRNAs, up-regulated in HFD SPZ **(A)** or down-regulated in HFD SPZ **(B),** whose host genes are located on different chromosomes, show tethering activity toward the same miRNA as target.

A

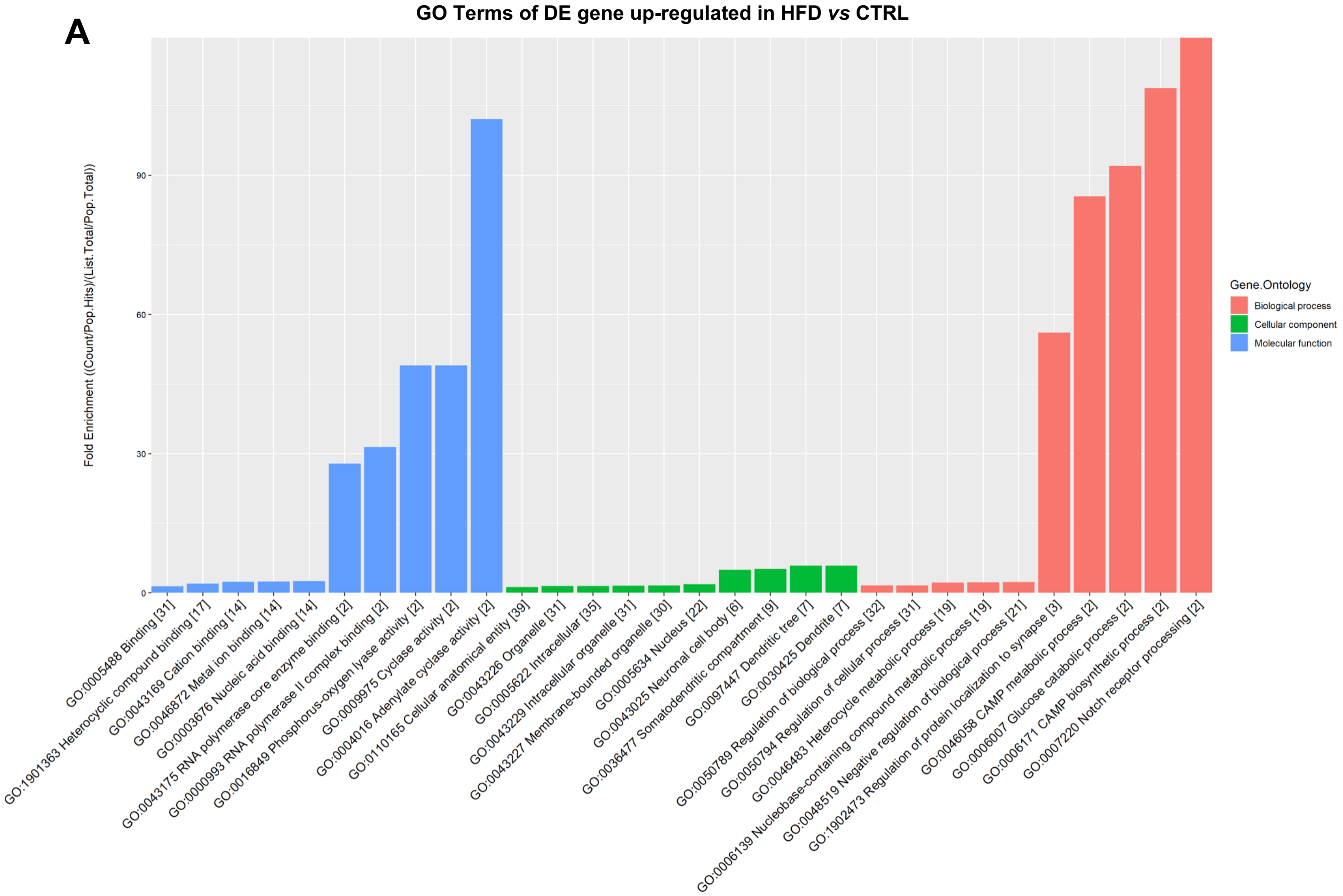

B

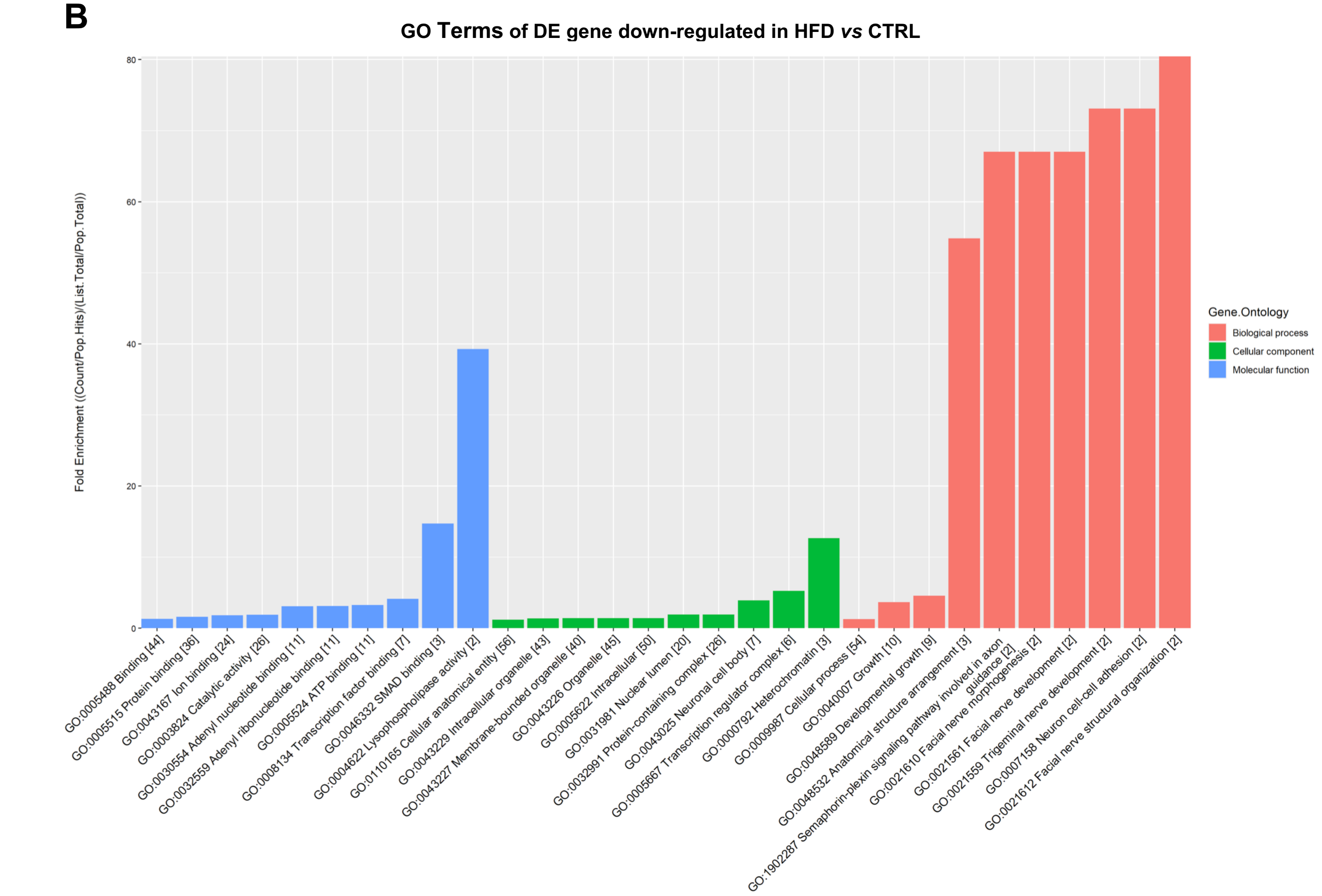

Figure S3. Gene ontology (GO) enrichment analysis of host genes. **(A)** The Top GO annotations in HFD SPZ up-regulated circRNAs. **(B)** The Top GO annotations in HFD SPZ down-regulated circRNAs.

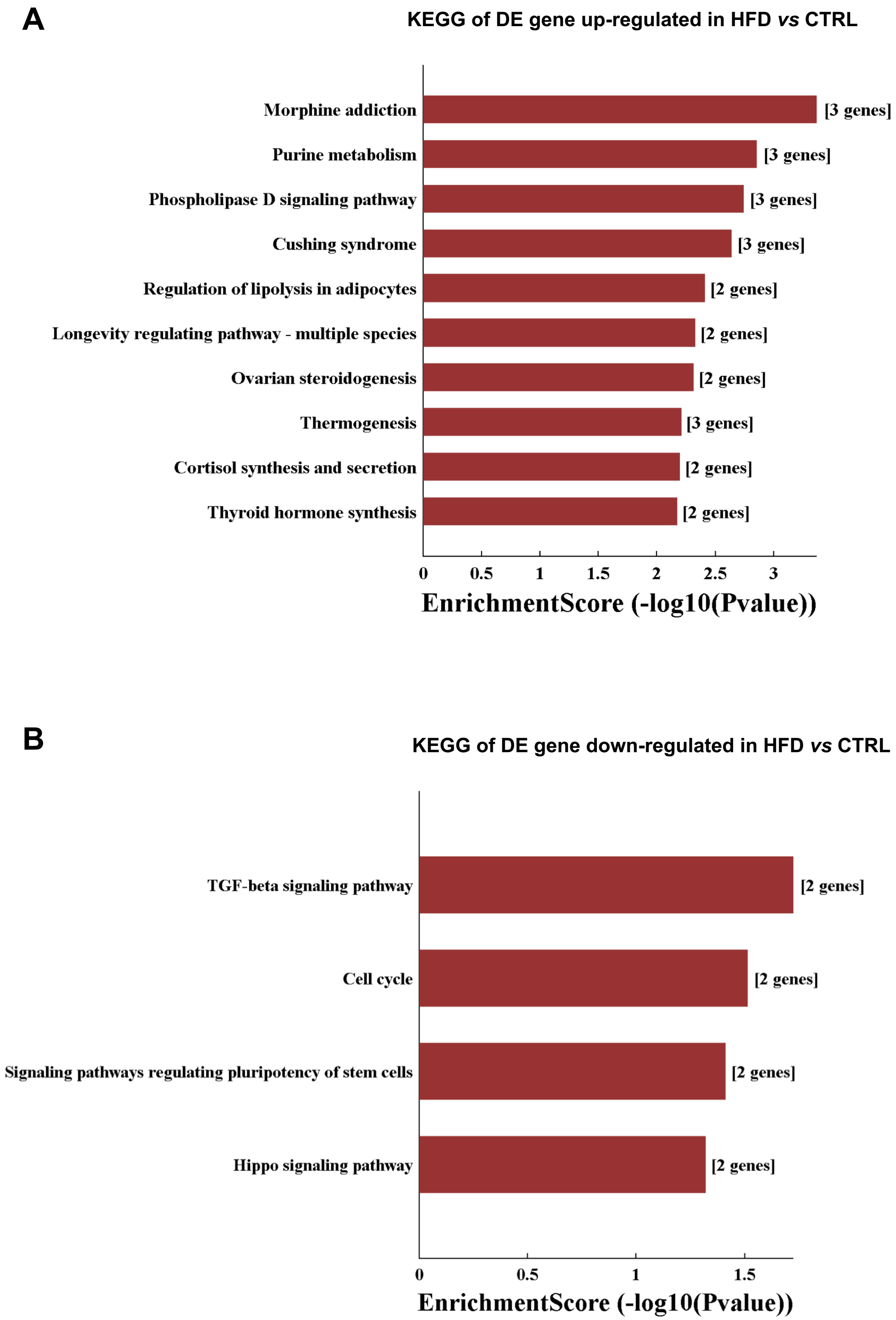

Figure S4. Kyoto Encyclopedia of Genes and Genomes (KEGG) pathway annotation of host genes. **(A)** The Top KEGG signalling pathway annotations in HFD SPZ up-regulated circRNAs. **(B)** The Top KEGG signalling pathway annotations in HFD SPZ down-regulated circRNAs.

A

CeRNET of a circRNA up-regulated in HFD *vs* CTRL SPZ

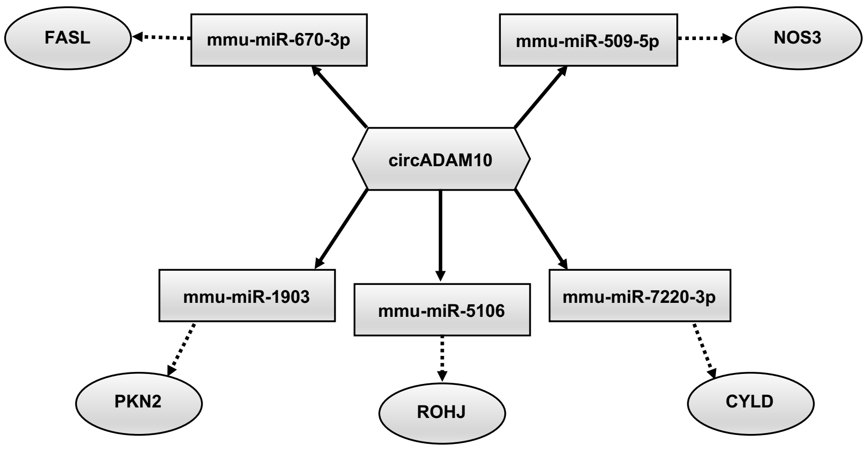

B

CeRNET of a circRNA down-regulated in HFD *vs* CTRL SPZ

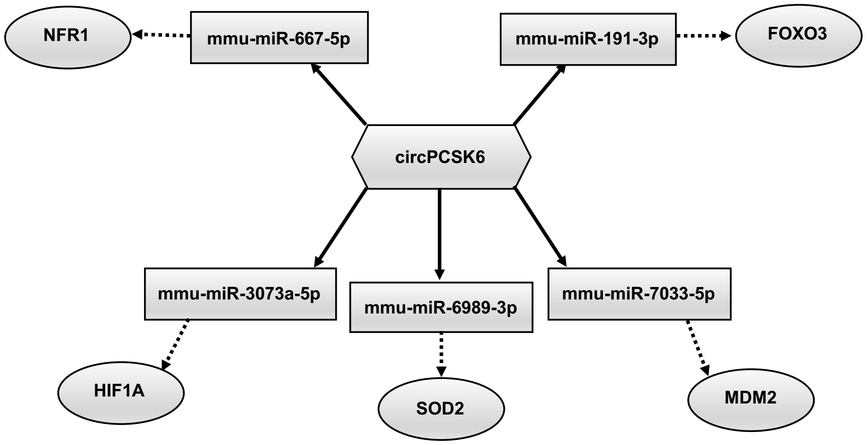

Figure S5. CeRNET of DE-circRNAs in HFD SPZ. (**A**) One circRNA up-regulated in HFD SPZ, circADAM10, and (**B)** one circRNA down-regulated in HFD SPZ, circPCSK6, tether a group of miRNAs as targets, all involved in oxidative stress pathways. Networks were built using Cytoscape. Hexagonal and rectangular symbols represent circRNAs and miRNAs, respectively. The arrow indicates the tethering activity of circRNAs toward miRNAs, while the dotted arrow indicates the pathways upstream of the miRNAs.

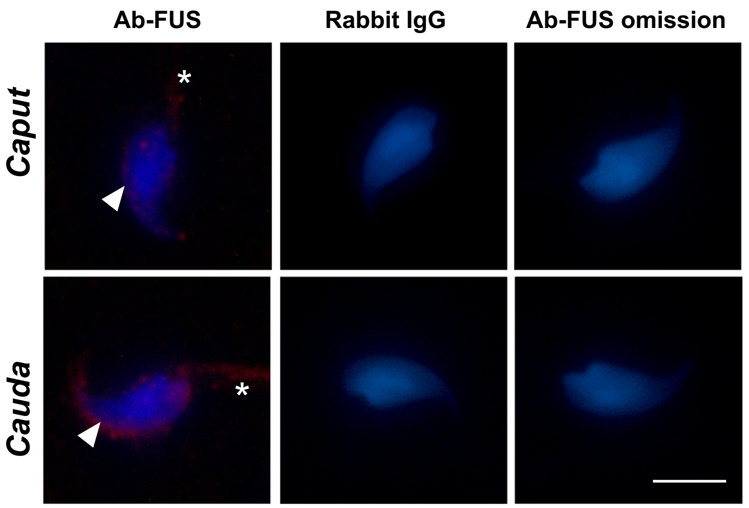

Figure S6. FUS immunofluorescence quality controls. Immunofluorescence analyses of Ab-FUS (red) and rabbit IgG (red) in *caput* and *cauda* SPZ. An additional negative control consisting of primary Ab-FUS omission was performed to avoid non specific binding of the secondary antibody. White arrowheads and asterisk represent FUS sperm head and tail localizations, respectively. Nuclei were labeled with DAPI (blue). Scale bar corresponds to 5 µm.
